# Resistance of ERG24 sterol C-14 reductase to heterocyclic amine antifungals

**DOI:** 10.1038/s44259-025-00155-7

**Published:** 2025-10-01

**Authors:** Corinne J. Arnold, Laetitia Chartrain, David M. Lawson, James K. M. Brown

**Affiliations:** 1 John Innes Centre, Norwich Research Park, Norwich, UK; 2 Present Address: Camena Bioscience, Chesterford Research Park, Cambridge, UK

**Keywords:** Antifungal agents, Fungal genetics, Agriculture, CRISPR-Cas9 genome editing

## Abstract

There is an urgent need for a wider range of antifungals in medicine and agriculture and for their dual use in both contexts to be minimised. As new modes of action may incur unforeseen side-effects, existing groups of antifungals which have had limited use may be attractive options for development. Here, we report mutations in ERG24 sterol C-14 reductase which cause resistance of wheat powdery mildew to heterocyclic amine fungicides with a phenylpropyl chain, and confirm their effect by single-base editing in yeast. The resistance mutations have at most small effects on mitotic fitness measured by yeast growth parameters. Predictive protein modelling indicates that phenylpropylamines likely obstruct the sterol substrate’s access to the catalytic site, and thus act as competitive inhibitors, but do not bind directly to the catalytic residues. This information may support structure-guided development of new antifungals targetting ERG24 as alternatives to the widely-used ERG11 inhibitors.

## Introduction

Control of chronic, systemic and severe fungal diseases in human and veterinary medicine relies heavily on antifungal drugs^1^, while fungicides are widely used in crop protection when plant breeding and agronomy do not provide adequate control of diverse pathogens^2^. Widely used antifungals attack a limited range of enzymes^3^, many of which affect sterol synthesis or composition. Much the largest and most widely-used group in both medicine and agriculture are inhibitors of lanosterol 14α-demethylase in the ergosterol biosynthesis pathway (ERG11, also known as CYP51^1–7^; pathway summarised in Supplementary Fig. 1^8^) while other targets include ERG1 (squalene epoxidase)^9^, ERG2 (∆7-cholestenol ∆8-∆7 isomerase)^10,11^, ERG24 (∆14-sterol reductase)^10,11^ and ERG27 (3-ketoreductase)^12^.

There is an urgent need for new antifungals with more diverse modes of action^1,2,13–19^. First, extensive exploitation of just a few modes of action has selected resistance in many pathogens, especially to single-site inhibitors^1,19^. In medicine, there is now widespread resistance of most invasive fungal pathogens to antifungals, especially azole inhibitors of ERG11^1,3,13–17,20^, and pathogens resistant to most or all licensed antifungals have emerged^3,13,14^. In agriculture, monoculture of crop species and cultivars to which fungicides are applied routinely has contributed to the rapid emergence of fungicide resistant genotypes^2,17^. Resistance to multiple fungicides is especially common in powdery mildews because of their high evolutionary potential^5,21^.

A second reason for developing new antifungals is that even those with the same mode of action can have differential efficacy against different pathogens, while pathogen mutations can have differential effects on resistance to similar antifungals^22,23^. A third, related driver of innovation is the need to minimise off-target effects, including harm to host species and the wider environment^17,24,25^. The widespread dual use of azoles in human and veterinary medicine but also wood preservation and crop protection is especially troublesome because azole-resistant fungi acquired from diverse settings can cause near-unmanageable infections, not only by the well-studied *Aspergillus fumigatus*^14–17,26,27^ but also other important pathogenic fungi^28–30^.

Although it is desirable to have access to antifungals with diverse modes of action, investment in inhibitors of new modes of action is challenged by regulatory issues^31^. Existing groups of antifungals may supply useful leads, especially when their use to date has been limited and when their modes of action are known and mechanisms of resistance are understood. This is exemplified by carboxin, which was introduced in 1969 to control smuts (Ustilaginomycotina) but became the forerunner of the large group of succinate dehydrogenase inhibitors used for broad-spectrum control of many ascomycete and basidiomycete pathogens in agriculture from 2003 onwards^32^. New compounds might be used as single-product formulations or in combination therapy with antifungals with other modes of action^3,16,17,20,33^. Mixing or alternation of fungicides with different modes of action is standard practice in agriculture to minimise evolution of resistance^34,35^.

**Fig. 1:**
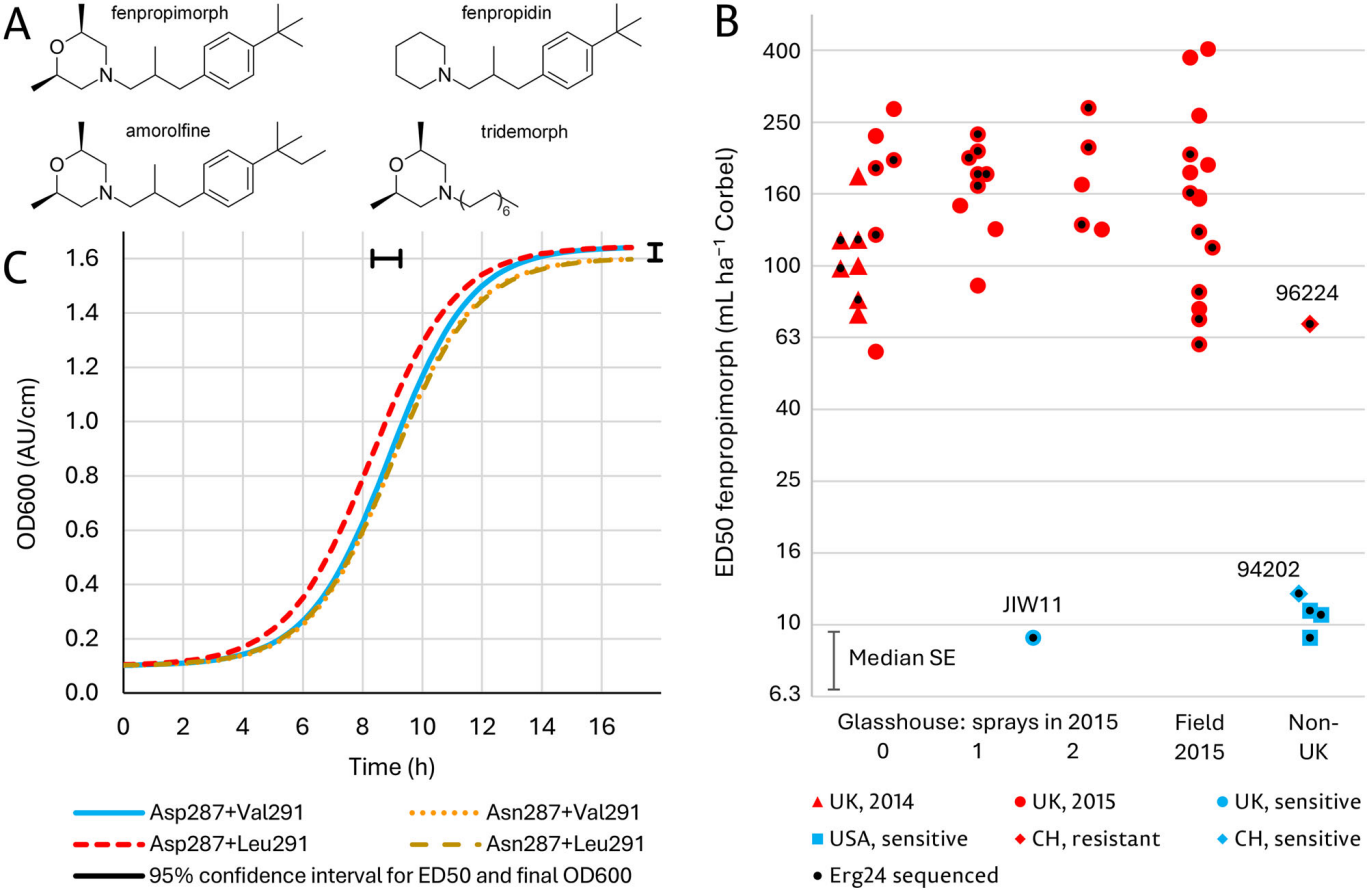
Structures of cyclic amine antifungals, wheat mildew responses to fenpropimorph and mitotic fitness of yeast strains with mutations in ERG24. **A** Structures of heterocyclic amine antifungals which inhibit ERG24 and ERG2. Structures are based on those in PubChem (https://pubchem.ncbi.nlm.nih.gov/). **B** Median effective doses (ED50) of Corbel (750 g L^−1^ fenpropimorph) for *Blumeria graminis* f.sp. *tritici* isolates sampled from John Innes Centre glasshouses in 2014 and 2015 and from the air spora above fields in Norfolk, England in 2015, together with reference isolates from the UK, Switzerland and the USA which were resistant or sensitive to phenylpropylamine antifungals. **C** Logistic growth curves of *Saccharomyces cerevisiae* S288C base-substituted strains in the absence of any antifungal. See Supplementary Table 6 for statistical analysis and parameter estimates. Confidence intervals for final OD600 and ED50 are shown, but the interval for the gradient was too narrow to be displayed (mean gradient = 0.274 +/− 0.003 h^−1^).

N-substituted heterocyclic amine antifungals have been used in both agriculture and medicine (Fig. 1A). Fenpropimorph, which was introduced in 1983 and has been the most widely used amine in agriculture, has a dimethylmorpholine ring with a *tert*-butylphenyl-methylpropyl (‘phenylpropyl’) sidechain. In tridemorph, introduced in 1969 as one of the first systemic agricultural fungicides, the dimethylmorpholine ring has an unbranched saturated aliphatic sidechain (C11-C14, mainly C13). Fenpropidin has a piperidine ring with the same phenylpropyl sidechain as fenpropimorph^10,36^. Amine antifungals have had limited use in medicine. Amorolfine, released in 1981, is structurally very similar to fenpropimorph. Currently the only amine antifungal with medical applications, it is used for topical treatment in dermatology, particularly onychomycosis^37^. Although amorolfine is well-tolerated^37–39^, a lack of systemic activity has precluded its use in internal medicine^39,40^.

Amine antifungals inhibit two enzymes, ERG24 and ERG2, which catalyse reduction or rearrangement of C:C double bonds in sterol synthesis^10,11,40,41^ and thus arrest fungal cell division^42^ (Supplementary Fig. 1). Among crop pathogens, resistance to amines has been studied in most detail in *Blumeria graminis*, which causes powdery mildew, a common disease of grasses and small-grain cereals, and has chalinasterol as its principal membrane sterol^43^ (Supplementary Fig. 2). Fenpropimorph and fenpropidin were highly active against mildew when first introduced but moderate resistance evolved during the 1980s^44^. Tridemorph was used from the 1960s to the 2000s; while it was less effective than the phenylpropylamines fenpropimorph and fenpropidin, only slight changes in sensitivity to it were observed even in barley mildew, its most significant agricultural target^44,45^. In striking contrast to important groups of broad-spectrum fungicides (azoles, quinone-outside inhibitors, succinate dehydrogenase inhibitors and benzimidazoles), complete resistance to phenylpropylamines has not evolved^5^ and they are still moderately effective in the UK against cereal powdery mildew and other target diseases^35^.

As with antibiotics^46^, information about target sites can support the development of new antifungals with a reduced risk of resistance, higher therapeutic value and fewer side-effects^3^. Similarly, mutations in target proteins can be exploited to gain insights into the mechanism of an antimicrobial. Here we propose the molecular basis for resistance of *B. graminis* to heterocyclic amines, involving point mutations in ERG24, V295L in wheat mildew and D291N in barley mildew (*B. graminis* f.sp. *tritici*: *Bgt* and f.sp. *hordei*: *Bgh*, respectively), homologous to V291L and D287N in *Saccharomyces cerevisiae*. As *B. graminis* cannot be transformed efficiently, the effect of these mutations on amine resistance was tested in *S. cerevisiae* by a CRISPR/Cas9 method of single base substitution. V291L in yeast caused resistance to phenylpropylamines and to a lesser extent tridemorph, while D287N caused a lower level of phenylpropylamine resistance but increased sensitivity to tridemorph. Both mutations had small effects on mitotic fitness of yeast but neither of them significantly altered responses to inhibitors of other steps in sterol biosynthesis. Predictive protein modelling suggested that amines impede access of the sterol substrate 4,4-dimethyl-cholesta-8,14,24-trienol (C29∆8,14,24) to the active site of ERG24. Although each antifungal molecule can adopt diverse poses within the access channel, they do not appear to mimic the sterol substrate or interfere directly with ∆14 reduction^47,48^ because the resistance mutations are not at catalytic sites. *Sc*Val291 (*Bgt*Val295) did not interact directly with the antifungal in any model while residue *Sc*Asp287 (*Bgt*Asp291) did so rarely. A completely conserved, non-catalytic residue, *Sc*Asp273 (*Bgt*Asp277), interacted with many poses across all antifungals tested. The combination of genetic evidence and structural predictions about the interactions of cyclic amines with ERG24 and mechanisms of resistance suggests options for investigation of new ERG24 inhibitors as alternatives to inhibitors of ERG11/CYP51.

## Results

### Detection of fenpropimorph-resistant wheat mildew

*Bgt* isolates were obtained from wheat mildew in glasshouses in 2014 which was not adequately controlled by fenpropimorph (as Corbel) or the azole prothioconazole (as Proline)^49^. Isolates fell into two groups by their median effective doses (ED50) of Corbel (Fig. 1B, Supplementary Table 1). The sensitive group (FPM-S) with five reference isolates had ED50 from 9.2–12.2 ml ha^−1^ (mean 10.4 ml ha^−1^) whereas the resistant group (FPM-R) had ED50 from 57.6–402 mL ha^–1^ (mean 145 mL ha^−1^), a 14-fold reduction in sensitivity to Corbel. ED50 of glasshouse isolates rose 1.6-fold between 2014 and 2015 but the number of Corbel sprays applied in 2015 only caused a slight increase in mean ED50. Isolates from the air spora near arable fields in Norfolk in 2015 had ED50 similar to those from glasshouses (Supplementary Table 2). *Bgh* was more sensitive to Corbel than *Bgt* as isolates DH14 and CC52, collected before the introduction of fenpropimorph, were sensitive even to the lowest dose (11 mL ha^–1^) while two more recent isolates, W4 and CC148, had ED50 of 10.7 and 29.8 mL ha^–1^, respectively (Supplementary Table 1).

### *Erg24* and *Erg2* sequences

Unlike the distantly related ascomycetes *Fusarium graminearum*^50^ and *A. fumigatus*^51^ only one *Erg24* gene was detected in *B. graminis* genome sequence data. *Erg24* sequences of *Bgt* isolate 96224 and *Bgh* isolate DH14 were identical to GenBank accessions EPQ63705.1 and CCU75655.1 from the same isolates, respectively. Both were 1595 bp long with two exons and one 55 bp intron, and there were 38 non-synonymous exon single-nucleotide polymorphisms (SNP), 106 synonymous exon SNP and 5 intron SNP between 96224 and DH14. The predicted ERG24 protein had 489 amino-acids and 10 predicted transmembrane (TM) segments, each comprised of one to three α-helices, as in the ERG24 homologue *Ma*SR1 of the methylotrophic bacterium *Methylotuvimicrobium* (formerly *Methylomicrobium*) *alcaliphilum*^52^. Protein sequences of ERG24 in *Bgt* and *Bgh* were 92.2% identical and the locations of TMs were substantially conserved (Supplementary Fig. 3).

All amino-acid variants at residues other than 295 were present in only one *B. graminis* form. In *BgtErg24*, all FPM-R isolates, including 96224, had a 938 G > C substitution relative to all FPM-S isolates, producing a V295L mutation in TM7. In the two more-resistant *Bgh* isolates, W4 also had 938 G > C while CC148 had 926 G > A resulting in a D291N replacement in TM7 of BghERG24. The only other protein sequence variation within forms concerned Y165F (549 A > T substitution) in 96224 and another FPM-R isolate relative to other *Bgt* isolates, and L77F (248 C > T) in W4 relative to other *Bgh* isolates. Homology of critical residues is summarised in Supplementary Table 3.

Across high-level taxa of eukaryotes, residues homologous to Asp291 and Val295 in *Bgt* were highly but not completely conserved (Supplementary Table 4). Exceptions were that representative species in Glomeromycota (vesicular-arbuscular mycorrhizae) and Mucormyceta, including “black fungus” mucormycosis pathogens, had Asn291. All Apomorphea (fungi, animals and relatives) had Val295 except the Glomeromycota species with Leu295, Diphoretickes (plants *sensu lato*) had Ile295 or Leu295 and the bacterium *M. alcaliphilum* had Leu295. By contrast, other residues in the sterol binding pocket were invariant throughout the tree of life, including several physically close to Asp291 and Val295 in TM5, 6 and 7.

In *Bgt* 96224, *Erg*2 was 799 bp long with three exons and two introns and encoded a 230-residue ERG2 protein identical to the GenBank sequence from 96224 (accession EPQ63078.1). There were three distinct coding sequences with a total of four synonymous SNP but no non-synonymous variation in predicted ERG2 sequences. No polymorphism in either *BgtErg24* or *BgtErg2* was associated with variation in Corbel ED50 among FPM-R isolates.

### Phenotypes of ERG24 mutations in yeast

As routine transformation of *B. graminis* is not yet possible, the effects of ERG24 mutations observed in *B. graminis* on responses to sterol biosynthesis inhibitors were tested by site-specific single nucleotide substitution by CRISPR/*Cas9* in the single-copy *Erg24* gene of yeast strain S288C^53^. The following mutations at *S. cerevisiae* residues Val291 (*Bgt*Val295) and Asp287 (*Bgt*Asp291) in wild-type (WT) ScERG24 were produced: V291L (three replicate strains, S288C-V291L-1 to V291L-3), D287N, a double mutant with D287N/V291L, a V291V dummy mutation, and a revertant (rev) with S288C-V291L-1 back-mutated to WT Val291.

**Fig. 2:**
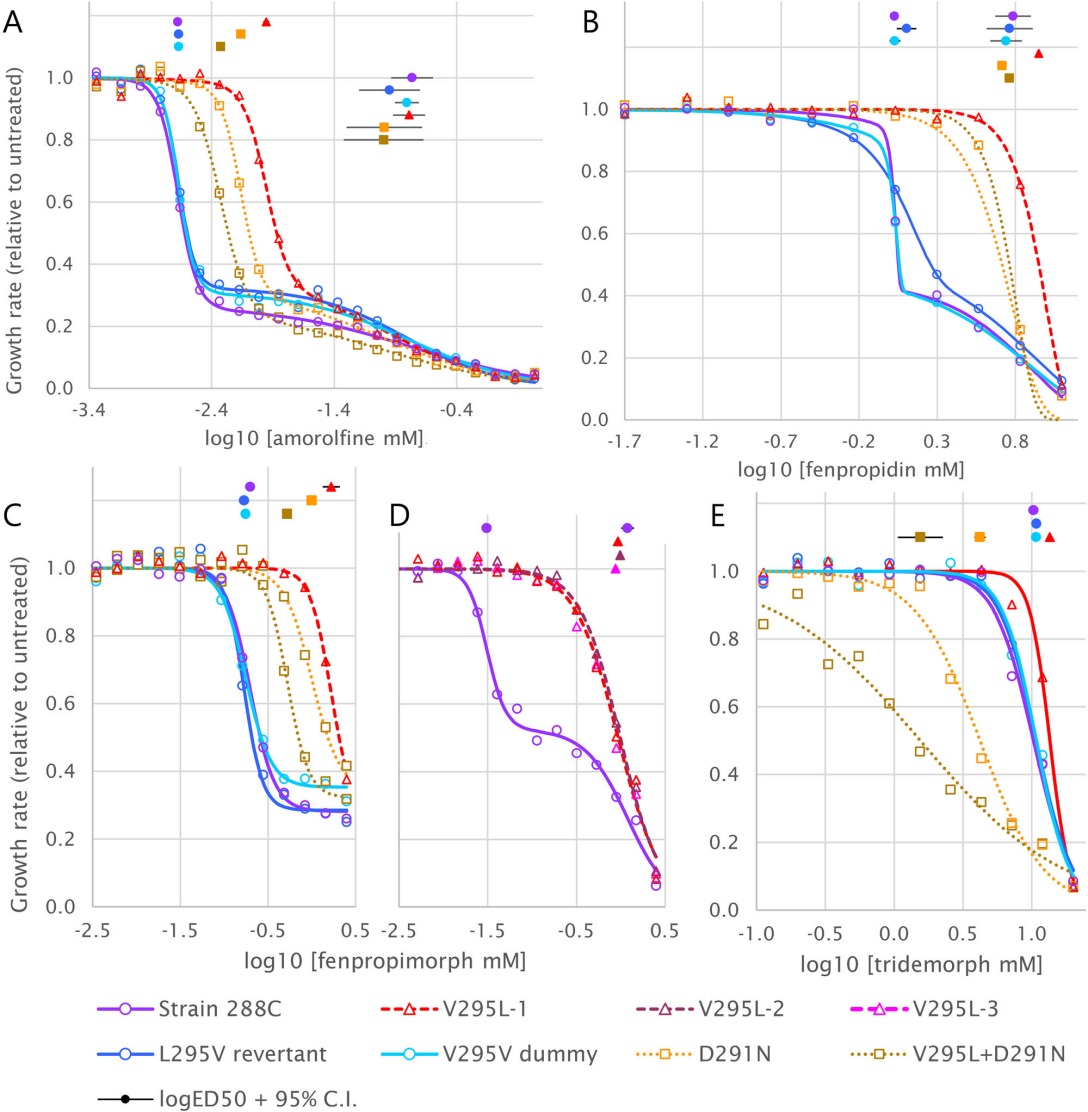
Responses of based-edited yeast strains to amine antifungals. Dose–response curves of yeast strain S288C with amino-acid substitutions introduced by single-nucleotide substitution using CRISPR/Cas9, showing variation in responses to amine fungicides **A** amorolfine, **B** fenpropidin, **C**, **D** fenpropimorph in two experiments, **E** tridemorph. Construction of yeast strains, the test method and curve-fitting procedures are described in ‘Methods’. Median effective doses (ED50) are shown together with 95% confidence intervals (CI); where the CI is not apparent, it did not extend beyond the marker for the ED50.

In all strains, the solvent-only treatment had no significant effect on fungal growth compared to water only. With all three phenylpropylamines, amorolfine, fenpropimorph and fenpropidin, S288C-WT had a two-phase response fitting a double logistic curve, consistent with these antifungals inhibiting two enzymes. S288C-V291V and S288C-rev, both with Val291, had responses to phenylpropylamines similar to S288C-WT for all three fungicides (Fig. 2A–D; Supplementary Fig. 4, Supplementary Table 5).

The V291L mutation increased the lower ED50 of amorolfine and abolished those of fenpropimorph and fenpropidin, implying it reduced or eliminated the responsiveness of the more sensitive target to phenylpropylamines. D287N also abolished the lower ED50 of fenpropidin but combined with wild-type Val291, had a smaller effect than V291L alone on the lower ED50 of amorolfine and fenpropimorph. In the D287N/V291L double mutant, the lower ED50 of amorolfine and fenpropimorph lay between those of S288C-WT and D287N. In all mutants, the higher ED50 was not significantly affected by either the D287N or V291L substitutions, implying no change in the response of the less sensitive target of phenylpropylamines (Fig. 2A–D; Supplementary Fig. 4, Supplementary Table 5). These data imply that the more sensitive response to these antifungals relates to ScERG24 and that the less sensitive target with the higher ED50 caused the residual sensitivity of V291L and D287N mutants.

Responses to tridemorph, which has a saturated aliphatic tail and principally inhibits ERG2 rather than ERG24^11^, contrasted with the phenylpropylamines. V291L slightly reduced sensitivity to tridemorph, increasing the ED50 relative to S288C-WT, but D287N increased sensitivity and the D291N/V295L double mutant increased it further (Fig. 2E; Supplementary Table 5).

Neither mutation significantly altered the sensitivity of S288C to terbinafine and ketoconazole, which inhibit ERG1 and ERG11, respectively (Supplementary Fig. 5, Supplementary Table 5). Effects on the ERG27 inhibitor fenhexamid could not be tested by this method because it is not toxic to *S. cerevisiae*^54^.

### Models of ScERG24 structure and ligand docking

**Fig. 3:**
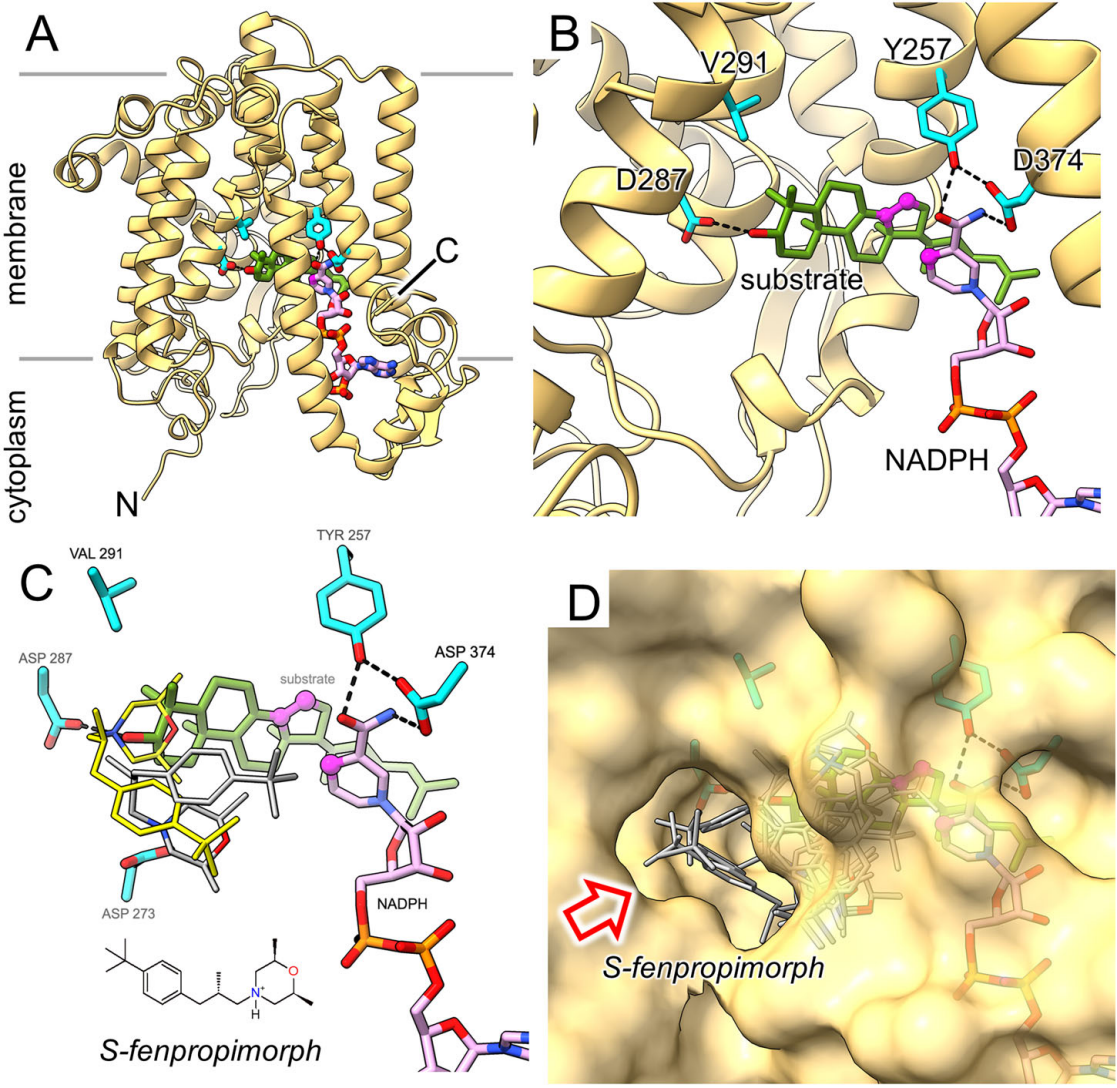
*Saccharomyces cerevisiae* S288C AlphaFold2 model of ERG24 and docking results. **A** Overall ERG24 model depicted in cartoon representation with its likely orientation in the membrane; key residues and ligands are shown in stick representation. **B** Close-up of putative active site region showing the predicted catalytic residues, Tyr257 and Asp374, the resistance-conferring mutation sites, Val291 and Asp287, and the conserved Asp273 that may play a role in fungicide binding (cyan carbons). The docked cofactor (pale pink carbons) and substrate (green carbons) are also shown. Highlighted in magenta spheres are the C4 atom of the nicotinamide ring in the cofactor and the C14:C15 double bond of the substrate. An α-helix in the foreground has been omitted for clarity; dashed lines indicate possible hydrogen bonds. **C** Same view as panel B with the protein cartoon removed, showing two of nine poses from a docking simulation with *S*-fenpropimorph, the more effective enantiomer^81^, performed in the absence of cofactor and substrate although both are shown here for reference. One pose is shown where the heterocyclic nitrogen (highlighted as a blue sphere) is poised to hydrogen bond to Asp287 (thin sticks; yellow carbons) and, in the other pose, the nitrogen is poised to hydrogen bond to Asp273 (thin sticks; grey carbons). All nine of the highest-ranking poses of each antifungal clustered near the mutation sites, with some partially overlapping the left-hand end of the substrate. A 2D representation of the *S*-fenpropimorph structure is shown as an inset. **D** Same view as (**C**) together with a semi-transparent molecular surface showing a potential substrate access channel (red arrow), which is obstructed by the docked fungicide molecules. The 3D structures are displayed using ChimeraX v1.8^80^ and the 2D sketch of *S*-fenpropimorph was produced using ACD/ChemSketch 2022.2.3.

ERG24 proteins from yeast strain S288C-WT, *Bgt* isolate JIW11 and *Bgh* isolate DH14 were modelled with AlphaFold2 (AF2^55^; Fig. 3). For the input sequences, the multiple sequence alignments showed very good sequence coverage, with several thousand matches obtained for each, this being a prerequisite for a thorough analysis of residue co-evolution in the AlphaFold2 algorithm. The five independent models generated for each protein were closely similar and all had high local confidence scores for the majority of the polypeptide, with pLDDT values exceeding 0.95 for the mutation sites and other residues predicted to be of importance. Similarly, the predicted aligned error (PAE) plots also showed high confidence in the relative placements of pairs of residues across the majority of the structures, with the exception of those in a few surface loops and at the chain termini, consistent with these regions being more flexible (Figs. S6–S8).

The AF2 models closely replicated major features of the crystal structure of the *Ma*SR1 enzyme in the methylotrophic bacterium *M. alcaliphilum*^52^, homologous to ERG24 in eukaryotes (Supplementary Fig. 9). As in *Ma*SR1, fungal ERG24 were predicted to have ten transmembrane (TM) segments and a channel formed by TM6, 7 and 10, with two openings which face into the same pocket within the channel. One opening, facing the cytoplasm, provides access for the NADPH cofactor, while the C29∆8,14,24 substrate may enter the holoenzyme through the other, facing the hydrophobic interior of the plasma membrane (Fig. 3A). Ring D of the sterol may thus interact with NADPH at the catalytic site (Fig. 3B). As hydrophobic molecules, the amine antifungals may gain access to the reaction pocket through the membrane-facing opening (Fig. 3D). In all possible pairwise comparisons of the three top-ranked AF2 models and the MaERG24 crystal structure, the overall root-mean-square deviations in Cα positions did not exceed 2 Å upon superposition and the key residues involved in catalysis and antifungal resistance overlapped closely, indicating a high degree of structural similarity (Supplementary Fig. 9). This establishes the validity of using ERG24 in *S. cerevisiae* to model the effects of mutations in *B. graminis* ERG24.

Plausible docking poses for NADPH in ScERG24 were selected with the adenine diphosphate portion of the cofactor bound in a similar fashion to the crystal structure of MaERG24. Additionally, the nicotinamide moiety of the cofactor was positioned such that the C4 hydrogen that is donated to C29∆8,14,24 was close to the predicted catalytic residues Tyr257 and Asp374 (Tyr261 and Asp282 in *Bgt*ERG24, Tyr241 and Asp363 in *Ma*SR1^52^) while allowing space alongside for the substrate to bind. In each run, there was a narrow spread of binding affinities (−30.6 to −29.2 kcal/mol) despite a wide variety of poses for the docked cofactor, so this was deemed not to be a suitable metric for choosing solutions. In two of the three runs, however, a single pose consistent with the above criteria was observed, one of which is presented (Fig. 3B).

Subsequently, the ScERG24 holoenzyme model including the NADPH pose in Fig. 3B was used as the receptor for docking runs with C29∆8,14,24. Again, there was a narrow spread of binding affinities (−9.0 to −8.0 kcal/mol), and suitable poses were selected such that the ∆14 double bond in sterol ring D was adjacent to both the hydroxyl of Tyr257 and the C4 hydrogen of NADPH. Similar poses were observed in replicate runs. Given uncertainties in the placement of key groups, it is not possible to propose a detailed hypothesis for the catalytic mechanism but the spatial arrangement of critical groups is consistent with the reduction mechanism proposed previously^48^, involving an electrophilic attack on the sterol double bond by the Tyr257 hydroxyl proton, possibly indirectly via a water molecule, and hydride transfer from the C4 nicotinamide hydrogen of NADPH^47^. This could be either as a concerted mechanism or stepwise via a carbocation intermediate). In this model, the role of Asp374 in catalysis could be to coordinate both the carbamoyl group of the cofactor nicotinamide moiety and the hydroxyl of Tyr257. In the plausible poses for C29∆8,14,24, ring A lies in the vicinity of the mutation sites and in some cases, the C3 hydroxyl forms a hydrogen bond with Asp287 (Fig. 3B) but there was no evidence that the sterol interacts directly with the other mutated residue, Val291.

In docking simulations with the antifungals, we explored the possibility that their mode of action could involve competitive inhibition of cofactor binding, sterol binding, or both. The antifungals were therefore allowed to sample potential docking sites of the ligand-free ScERG24 apoprotein model in a comparatively large volume encompassing the predicted binding sites for the sterol and the nicotinamide moiety of the cofactor, as well as the catalytic residues Tyr261, Asp382 and Arg406, and the mutation sites Asp287 and Val291. Visualisation of all the antifungal dockings in the context of a molecular surface for ScERG24 revealed that most were clustered at the inner end of the channel leading to the lipid bilayer (Fig. 3D). In the great majority of simulations with all four amines, the poses clustered near the two mutated residues Asp287 and Val291, and partially overlapped the position of sterol ring A in the ternary complex and occasionally clashed with it (Fig. 3C). Among the latter poses were a single pose for each phenylpropylamine, but not tridemorph, where the heterocyclic N was H-bonded to Asp287, with the dimethylmorpholine moieties of amorolfine and fenpropimorph in the same orientation overlapping the sterol site. In these single poses, the antifungal partially overlaps the predicted binding site of the sterol substrate and thus competes directly with it for binding to ERG24, but they were amongst the least favourable for each simulation ranked by binding energy. By contrast, for all four antifungals, there were at least three poses in the top five where the heterocyclic nitrogen hydrogen-bonded to Asp273 (*Bgt*Asp277, *Ma*Asp257), a fully conserved residue (Supplementary Fig. 4), but most of these did not clash directly with the sterol (Supplementary Fig. 10). No pose of any of the four antifungals was sufficiently close to Val291 to suggest a direct effect of a V291L substitution on docking, and poses overlapping with the predicted position of NADPH were rare. The residues homologous to the other two sites where substitutions were found in *B. graminis*, Tyr76 (Leu77 in *Bgh*, in TM2) and Tyr161 (Tyr165 in *Bgt*, TM4), were not located in the predicted sterol, cofactor or antifungal binding sites of ScERG24 and are thus unlikely to be involved in catalysis or amine resistance.

### Effect of ScERG24 mutations on yeast fitness

Mutations to amine resistance altered some yeast growth parameters relative to the dummy-edited strain S288C-V291V. The D287N mutation reduced final OD600 by 2.7% (*P* = 0.01), V291L reduced the growth rate parameter by 2.7% (*P* = 0.05) and the point of inflection of V291L-1 was 6.7% earlier (*P* = 0.02) (Fig. 1C; Supplementary Table 6).

## Discussion

There is an urgent need for more antifungals with more diverse modes of action to control diseases of people, domesticated animals and crops, and for timber preservation^1,13,15,16,19^. From the One Health perspective, it would be desirable for antifungals with different modes of action to be used in each of these contexts. Heterocyclic amines have been used against specific fungi in crop protection^5^ and dermatology^37^ but not as broad-spectrum agricultural fungicides or systemic antifungals in medicine. As an established group with high activity against a limited range of diseases, knowledge of the interactions of amine antifungals with ERG24 may support the design of new molecules with higher therapeutic value, broader activity against diverse fungi, fewer off-target effects and reduced risk of resistance. Improved ERG24 inhibitors would complement the heavily-used azoles that inhibit ERG11. This paper identifies amino-acid replacements that cause resistance of ERG24 to amines, shows they have limited effects on fungal fitness, provides structural information which supports an existing model of sterol reduction, suggests a new model for the antifungals’ mode of action, and proposes a mechanism of resistance. Furthermore, we demonstrate the value of base editing by CRISPR/Cas9 to test hypotheses about molecular evolution of antimicrobial resistance.

Resistance of *B. graminis* to phenylpropylamines involves mutation of ERG24 rather than their other target enzyme, ERG2. Fenpropimorph resistance in *B. graminis* was associated with D291N and V295L mutations in ERG24 but there was no protein sequence variation in ERG2. This was confirmed by recreating homologous site-specific single-nucleotide substitutions in ERG24 of yeast using CRISPR/*Cas9*, which reproduced the major effects of the two mutations in causing resistance to phenylpropylamines relative to both wild-type S288C *S. cerevisiae* and the dummy-edited V291V strain, while maintaining sensitivity to inhibitors of ERG11 and, in contrast to earlier reports^56,57^, ERG1. We propose *Bgt* as the archetype species for ERG24 phenylpropylamine resistance in the established system of numbering orthologous mutations to antifungal resistance^58^.

The increase in mean ED50 of Corbel between 2014 and 2015 was not associated with additional variation in ERG24 or any variation in ERG2, implying that as with azoles^49^, genetic variation at more than one locus must be involved in phenylpropylamine resistance. At least two genes were involved in fenpropimorph resistance in *Bgh*^59^, *Aspergillus niger*^60^ and *Ustilago maydis*^61^.

Amine resistance in *Bgt* conferred by D291N and V295L is incomplete. The extent to which ERG24 variation can reduce sensitivity to phenylpropylamines is presumably limited because these antifungals also attack ERG2^11^. The proposed mechanism of ERG2 inhibition, involving the dimethylmorpholine ring^11^, would also apply to amorolfine but not the piperidine derivative fenpropidin^45,59^. The existence of two targets may have contributed to the durable, partial effectiveness of fenpropimorph in agriculture^5,35^. Single-base substitutions in ScERG24 caused small changes in asexual growth compared to the dummy-edited V291V strain, which is an important component of fungal fitness. Measurement of fitness penalties of fungicide resistance has been challenging^4,62^ but here, the spectrophotometric assay of yeast growth was sufficiently precise to estimate the fitness component with small standard errors. If there is a fitness cost of resistance in *B. graminis*, it might have contributed to the continuing moderate effectiveness of phenylpropylamines but any such cost has evidently not been high enough to prevent the V295L mutation reaching a high frequency. Extrapolating to medicine, even if the fitness cost of resistance mutations were low in human and veterinary pathogens, the existence of a second target might increase the durability of amine antifungals.

Modelling of ERG24 in yeast followed by in silico docking provided insight into the mechanism of sterol reduction. It was previously reported that ∆14 reduction by ERG24 takes place by protonating C15 in the sterol D ring followed by hydride transfer to C14 from NADPH^47^. Our AF2 models support this mechanism, suggesting that the ∆14 double bond is reduced by the *Bgt*Tyr261 hydroxyl group and that hydride transfer is from C4 of the nicotinamide ring in NADPH. The models are largely similar to the crystal structure of *Ma*SR1, although the modelling of the ternary complex in that enzyme placed the sterol substrate in an orientation such that the ∆14 bond was not close to the corresponding catalytic residues or the nicotinamide ring^52^.

Detailed hypotheses about molecular interactions between antifungals and ERG24 should be treated with caution as they are based on predictive modelling, so we focus on robust conclusions supported by genetic data and visualised in multiple poses. Each antifungal, when docked into the AF2 model of *Sc*ERG24, assumed diverse poses with no strong preference for any single pose. Nevertheless, in virtually all poses, the antifungal compound would either impede access of the C29∆8,14,24 sterol substrate to the catalytic site by blocking the channel leading from the membrane, or prevent formation of a catalytically competent enzyme substrate complex by overlapping with the substrate binding site. In contrast to a previous proposal, our models gave little support to the hypothesis that the *N*-heterocycle mimics a sterol ring within the catalytic site^47^. Tridemorph, with a long aliphatic side-chain, is much less toxic to ERG24 in WT yeast than fenpropimorph despite having the same dimethylmorpholine ring^10,11^. Although the saturated, unbranched aliphatic chain of tridemorph is similar in size to the more rigid, bulky phenylpropyl group, its flexibility may make it less effective at obstructing the sterol.

It is possible that the diversity of poses of phenylpropylamines within the active site reflects a similar variety *in fungo*. If so, the mechanism of resistance may involve altered competition between the sterol and antifungals for presence within the catalytic pocket rather than interference with a specific protein-ligand interaction. In our models, neither of the mutated residues *Bgt*Asp291 and *Bgt*Val295 were close to the catalytic site, there was no evidence that *Bgt*Val295 interacted directly with either the sterol or the antifungals, and in only a few poses was *Bgt*Asp291 close enough to either the sterol C3 hydroxyl or the antifungals’ heterocycle N to H-bond with them. Nevertheless, replacement of these amino-acids caused complete or high resistance of ERG24 to phenylpropylamines. We suggest that in WT ERG24, the phenylpropylamines may outcompete the sterol substrate C29∆8,14,24 whereas the reverse may be the case in the *Bgt*V295L and *Bgt*D291N mutants, so the antifungal inhibitor is displaced. In contrast, *Bgt*D291N might increase affinity for tridemorph relative to the sterol, thus reducing its ED50. It is conceivable that the mutations might have other effects on the function of ERG24 in the presence of the antifungal, but we have not explored additional processes such as release of the sterol product or protein stability.

This mechanism of resistance accords with the genetics of amine resistance in *Bgh*, in which one gene conferred resistance to both fenpropimorph and fenpropidin, which have the phenylpropyl sidechain in common but different heterocycles, but not tridemorph, although it shares the dimethylmorpholine ring with fenpropimorph^59^. Presumably this gene was *Erg24*. In a population of *Bgh* in Hampshire, England in 1990, there was negative cross-resistance between tridemorph and phenylpropylamines^45^, as in the base-edited strains reported here. Speculatively, this could have happened if phenylpropylamine resistance there had involved D291N as in *Bgh* isolate CC148, so application of tridemorph or phenylpropylamines had selected Asp291 or Asn291, respectively. Possible variation in resistance mechanisms should be considered in DNA-based surveys of fungicide resistance.

It is intriguing that in many poses, the antifungal heterocycle N was H-bonded to a fully conserved residue of ERG24, *Bgt*Asp277. This residue is not close to the mutated amino-acids and is at the opposite end of the channel from the catalytic residues *Bgt*Tyr261 and *Bgt*Asp382. The invariance of *Bgt*Asp277 throughout the tree of life and the toxicity of antifungals which are predicted to bind to it imply that this amino-acid is essential for the function of ERG24 but its role is not apparent at present.

Low specificity of existing amines to ERG24 in fungal pathogens relative to their hosts has limited their value as systemic antifungals in medicine^39^ and broad-spectrum fungicides in crop protection^47^. Attempts to increase the efficacy of phenylpropylamines against crop diseases focussed on the structure of the tail of the antifungal molecule but were unsuccessful^47^. This approach might have been unproductive because of the variable location of the phenylpropyl group within the sterol access channel. Insights into the interaction of amine antifungals with ERG24 may provide opportunities to design new molecules with increased efficacy and specificity, interacting with residues which are synapomorphic in fungi relative to plants or animals. These include several sites in the TM6-7 linker containing the invariant *Bgt*Asp277 (Fig. 3c, Supplementary Table 9, Supplementary Fig. 10). Exploiting structural analysis for structure-guided design of new antifungals may help to increase the value of ERG24 inhibitors. If more effective phenylpropylamines were available for internal medicine, where few alternatives are currently available, these antifungals could be reserved for medical use, thus lessening the scope for dual use of molecules with the same mode of action in crop protection and timber preservation.

## Methods

### Fungal isolates

Responses to heterocyclic amine antifungals were studied in *Blumeria graminis* f.sp *tritici* (*Bgt*) and f.sp. *hordei* (*Bgh*), the powdery mildew pathogens of wheat and barley, respectively (Supplementary Table 1). In 2014 and 2015, wheat mildew in glasshouses at the John Innes Centre was not fully controlled by Corbel (active ingredient fenpropimorph 750 g L^−1^; BASF). Corbel had been used occasionally at JIC since 1990 and was used twice in 2015. Mildew-infected leaves were collected from glasshouse wheat plants in November 2014 and early 2015 before the first spray of Corbel in 2015, then after each spray that year. 27 *Bgt* single-spore isolates from different plants were used in the work reported here. A further 15 *Bgt* isolates were collected from mildew colonies on seedlings of the susceptible wheat cultivar Cerco exposed to the air spora^63^ at rural locations in Norfolk in 2015. The Swiss *Bgt* isolates 96224 and 94202 were kindly provided by Prof. Beat Keller, University of Zürich and three US *Bgt* isolates by Dr Christina Cowger, USDA-ARS and North Carolina State University^64^. *Bgt* isolate JIW11 was obtained from JIC’s *B. graminis* collection^65^, making 32 *Bgt* isolates in total. *Bgh* isolates DH14, CC148 and CC52 were also obtained from JIC’s collection^65^ while W4 was collected from the air spora on the susceptible barley cultivar Golden Promise in 2017. 96224 and DH14 are reference isolates for genomic research on *Bgt*^66^ and *Bgh*^67^, respectively.

### Fungicide spray tests

Sensitivity of *B. graminis* isolates to fenpropimorph was measured as the median effective dose (ED50) by a spray application method^45^. Seedlings of Cerco wheat and Golden Promise barley were used for *Bgt* and *Bgh* spray tests, respectively. For *Bgt*, a series of nine doses of Corbel at 1.9-fold intervals up to the recommended field rate (11, 21, 40, 77, 146, 277, 526 and 1000 mL ha^−1^) was applied plus water as a 0 mL ha^−1^ treatment. For *Bgh*, seven doses at 1.9-fold intervals were used (0, 5.8, 11, 21, 40, 77 and 146 mL ha^−1^). Leaf segments inoculated with mildew were incubated at 15 °C and sporulating colonies were counted on each segment after 8 days for *Bgt* and 7 days for *Bgh*.

The median effective dose (ED50) of Corbel for each isolate was calculated by fitting a logistic curve to data on mildew colony numbers against log_10_(dose), with a Poisson distribution of colony counts. First, a logistic curve was fitted separately for each isolate but the very steep slope of the dose-response, which causes high variance of mildew colony number around the ED50, caused the estimates of ED50s to be imprecise. The median of the slope parameters for each isolate was therefore used as a common slope parameter for all isolates. To avoid undefined values, log_10_(dose) at the zero dose was set to a value twice as far below the lowest non-zero dose on the log_10_ scale as the interval between the lowest two non-zero doses. Isolates were tested in four batches so a single ED50 was estimated for each isolate by fitting a linear model of Batch + Isolate to the ED50 estimates, weighted by the inverse of the standard error of each estimate. Statistical analysis was done with Genstat edition 24 (VSN International).

### Sequencing *Erg24* and *Erg2*

Primers were designed for PCR amplification and sequencing of the complete *Erg24* gene in *Bgt* (reference isolate 96224, assembly scaffold 39, GenBank accession KE375101.1) and *Bgh* (reference isolate DH14, assembly scaffold sca005483, accession HF943546.1), and the complete *Erg2* gene in *Bgt* (assembly scaffold 49, accession KE375133.1) and *Bgh* (assembly scaffold sca005449, accession: HF943512.1) in order to identify mutations present in isolates with reduced sensitivity to fenpropimorph (Supplementary Table 7, which also contains Genbank accession numbers). PCR amplification was done with Q5 polymerase (New England BioLabs Inc., UK) with the following conditions: initial denaturation at 98° for 30 s followed by 33 cycles of denaturation at 98° for 10 s, annealing for 20 s and elongation at 72° for 50 s, then a final extension at 72° for 120 s followed by holding at 4°. Annealing temperatures were 66° for Bgt Erg24, 64° for Bgh Erg24 and 68° for Bgt Erg2. All PCR products were purified using a QIAGEN QIAquick PCR purification kit following the manufacturer’s instructions. DNA was quantified using a NanoDrop2000 spectrophotometer, diluted appropriately and sequenced using Sanger sequencing by Eurofins Genomics (https://eurofinsgenomics.eu/). Sequences were trimmed and aligned to the sequences of the reference isolates using Vector NTI ContigExpress (Thermofisher).

Sequences of ERG24 from *Bgt* isolate JIW11, *Bgh* isolate DH14, *Saccharomyces cerevisiae* S288C and *Methylotuvimicrobium alcaliphilum* in Supplementary Fig. 3 were aligned by Clustal Omega v1.2.4^68^ then displayed using ESPript3.0^69^ (http://espript.ibcp.fr/ESPript/ESPript/). ERG24 sequences in Supplementary Table 4 were aligned by MUSCLE 3.8.425^70^ on the EMBL-EBI server. Data on principal membrane sterols in Supplementary Table 4 were obtained for Eumycota^71,72^, *Dictyostelium discoideum*^73^, wheat^74^, red and brown algae^75^, Leishmania^76^ and Methylococcaceae^77^.

### ERG24 mutations in yeast

The wild-type (WT) reference strain of *Saccharomyces cerevisiae*, S288C, was obtained from the National Collection of Yeast Cultures, Quadram Institute of Bioscience, Norwich, UK. Single nucleotide substitutions in the sequence of the WT *ScErg24* gene, available at https://www.yeastgenome.org, were engineered by a CRISPR/Cas9 method^53^. Three 20 bp guide sequences were designed to produce a V291L mutation in WT ScERG24 to test the effect of the homologous V295L mutation in BgtERG24, while one guide sequence was designed to generate a D287N mutation in WT ScERG24, homologous to D291N in BghERG24. In each case, a synonymous mutation was introduced in the guide sequence to enable validation of the transformation method. A double mutant (D287N + V291L) was produced by mutating the V291L-1 strain with the D287N guide. A dummy mutant (V291V) was created using the V291L-1 guide but mutating the GTT codon for *S. cerevisiae* base 291 to GTC, resulting in a synonymous mutation at residue 291. For the L291V revertant, a guide and repair template were designed to revert the Lys codon (CTT) at base 291 in strain V291L-1 and the Ser codon (TCA), a synonymous mutation at base 305, to the wild-type sequences GTT at base 291 and Serine (TCC) at base 305, respectively (Supplementary Table 8a).

All guide sequences were cloned into the pCas #60847 plasmid (Addgene) following the published method^53^ with minor modifications, using a 20 bp overlap with the repair oligonucleotide instead of 10 bp in the original protocol. DNA from putative mutants was extracted using the MasterPure^TM^ Yeast DNA Purification kit (Epicentre) following the manufacturer’s instructions. Correct integration was tested by PCR and sequencing (Supplementary Table 8b).

### Yeast growth assays

Antifungals used in yeast growth assays were obtained as pure formulations from Merck Life Science (amorolfine, fenpropidine, terbinafine, ketoconazole and tridemorph) and Cayman Chemical (fenpropimorph). All antifungals were dissolved to 1 M in an appropriate solvent (fenpropimorph in ethanol, fenpropidin and tridemorph in acetone, terbinafine in methanol) except amorolfine hydrochloride, which was dissolved to 100 mM in DMSO, and ketoconazole, dissolved to 500 mM in methanol. Stock solutions were then diluted to the highest dose tested which varied with each fungicide. The highest dose was serially diluted to obtain 11 or 21 doses as required. In each experiment, water was used as the 0 mM dose, and a solvent-only control treatment was included by adding the relevant solvent at its concentration in the highest antifungal dose.

For growth assays, yeast cells were grown overnight in 10 mL YPD medium at 28 °C then harvested and diluted in YPD to give an OD of 0.1 at 600 nm measured by spectrophotometry (BioPhotometer, Eppendorf). Yeast was then grown to OD 0.2 before fungicides were added at the appropriate concentrations in 96-well plates (Nunc 96 well cell culture plates, Thermofisher). Each concentration of antifungal was tested in triplicate with each strain, and each well was inoculated with 100 µl of cell suspension and 100 µl antifungal solution. Yeast growth was measured as the difference between OD at 600 nm (OD600) at 0 and 20 h (T0, T20), with plates incubated at 28 *°*C, measured with a FLUOStar Omega Microplate Reader, BMG Labtech. The low solubility of the antifungals generated minor technical issues. At the highest dose of amorolfine and terbinafine, OD600 values at T0 were considerably higher than the expected value of 0.1 because the fungicide solution became cloudy when the yeast cells were added. After 20 h incubation, the solution had cleared and OD600 values were low, as expected for doses well above the ED50. To set appropriate OD600 values at T0 for these cells in order to calculate fungal growth, the average OD600 value at T0 across all doses (minus the cloudy dose) for the relevant genotype was calculated and used as the baseline value of OD600 at T0.

The ED50 of each antifungal for each strain was estimated by non-linear modelling of the curve of fungal growth against log_10_(dose). For the zero dose, log_10_(dose) was set to a value twice as far below the lowest non-zero dose on the log_10_ scale as the interval between the lowest two non-zero doses. A range of models was examined and for each antifungal, one type of model clearly had the best fit as indicated by the coefficient of determination (*r*^2^) and was fitted for all strains: Gompertz curves for fenpropidin, rectangular hyperbolae for ketoconazole and logistic curves for all other fungicides. The ED50 was calculated from the fitted growth curve as the dose which reduced mean growth by half. For some strains and fungicides, double logistic or double Gompertz curves were fitted and ED50s calculated for each part of the curve (Supplementary Table 4). Each fungicide was tested in three to six replicate experiments and the graphs presented here are from those which included the largest number of yeast strains. Data from different replicates could not be combined because the non-linear parameters could not be estimated from multiple datasets.

Growth curves for base-edited yeast strains were obtained in the absence of fungicide to investigate fungal fitness in terms of asexual reproduction. For WT S288C and the V291L-1, D287N and dummy-edited V295V strains, replicate cultures of each strain were tested on nine dates with three technical replicates per culture, while the V291L + D287N strain was tested with six replicate cultures. OD was measured every 30 min over 20 h for each technical replicate. Mean parameters of the logistic growth model were calculated for each strain by linear mixed modelling with strain as a fixed effect and date, culture within strain and technical replicate within culture as random effects (Supplementary Table 5).

### Modelling ScERG24 structures and ligand docking simulations

In order to put our experimental observations into a three-dimensional context, we used a combination of structure prediction and in silico docking. The structures of ERG24 from *Saccharomyces cerevisiae* strain S288C, *Bgt* isolate JIW11) and *Bgh* isolate DH14 (Genbank accession numbers in Supplementary Table 4) were predicted using AlphaFold2^55^, as implemented through Colabfold v1.5.2^78^ (https://colab.research.google.com/github/sokrypton/ColabFold/blob/main/AlphaFold2.ipynb) and subsequently relaxed with Amber force fields; in other respects the default Colabfold settings were used. The top-ranked ScERG24 model was selected for in silico docking of substrates and inhibitors using AutoDock Vina 1.1.2 with default parameters, except for setting an exhaustiveness value of 32 and a hydrogen bond weight of −1.8. For the antifungal docking runs, 20 rather than the default 9 poses were requested. Each simulation was performed in triplicate, starting from a random seed each time. Input coordinates for all ligands were generated using AceDRG v249^79^. All possible rotatable bonds in the ligands were allowed to rotate freely during the simulations, but since the conformations of the various ring systems cannot be optimised as part of the docking process, we selected extended low energy conformations for each of them.

The NADPH cofactor was docked first into a box of dimensions 28 × 34 × 32 Å, which encompassed the equivalent region to that occupied by the partially resolved cofactor in the crystal structure of MaERG24 from *Methylomicrobium alcaliphilum*^52^ (named MaSR1 in ref. ^52^; PDB code 4QUV), as well as the region around the putative catalytic residues, Tyr257 and Asp374 in ScERG24, where the nicotinamide moiety would be expected to bind. The docking simulation was optimised by allowing flexibility in the side chains of residues likely to be involved in cofactor binding, namely: Met222, Trp225, Tyr257, Met271, Phe328, Asn332, Lys335, Arg339, His368, Asn370, Tyr371, Asp374, Ile377, Trp381, Phe399, Arg406, Asp410, Lys413, Lys417 and Tyr418. Plausible poses were selected such that the adenine diphosphate portion of the cofactor was bound in a similar fashion to that seen in the crystal structure of MaERG24 (in the latter the nicotinamide moiety is not visible) and the nicotinamide moiety of the cofactor was positioned with the C4 hydrogen, which is donated to the substrate by hydride transfer, close to the predicted catalytic Tyr257, but allowing space alongside for the substrate to bind.

The binary complex between ScERG24 and the favoured NADPH docking pose was then used as the receptor for docking simulations with the substrate, 4,4-dimethyl-5α-cholesta-8,14,24-trien-3β-ol (C29∆8,14,24). This time the docking volume included the active site residues and an adjacent cavity, having dimensions of 16 × 14 × 18 Å, with the side chains of Glu217, Met222, Tyr257 and Met271 allowed to be flexible. The sterol substrate was bound with the C and D rings approximately parallel to the nicotinamide ring and with the C14-15 double bond to be reduced near both the Tyr275 hydroxyl and the C4 hydrogen donated by the cofactor and within approximately 4 Å of both of them.

The antifungals were separately docked into the ligand-free ScERG24 model without flexible side chains, using a search box of dimensions 26 × 26 × 26 Å covering the predicted binding sites for the substrate and the nicotinamide moiety of the cofactor, as well as the catalytic residues and the mutation sites Asp287 and Val291. In running these simulations, we assumed that the antifungals were all protonated at the heterocyclic nitrogen, as previously suggested at physiological pH^11^. Docking was performed with both stereoisomers of fenpropimorph, fenpropidin and amorolfine, and the non-chiral tridemorph.

All protein structure figures were prepared using ChimeraX v1.8^80^ and 2D sketches of the fungicides were produced using ACD/ChemSketch 2022.2.3.

## Supplementary information

Arnold & Chartrain ERG24, Supplementary material

Arnold & Chartrain ERG24, Supplementary data

## Data Availability

Fenpropimorph response data for *B. graminis* and data on yeast growth are available at 10.5281/zenodo.15412169. *Erg24* and *Erg2* gene sequences have Genbank accession numbers PQ848564 to PQ8485671.

## Code availability

Genstat code for modelling double-logistic dose-response curves is available at 10.5281/zenodo.15412169.
