# Supplementary material for: Resistance of ERG24 sterol C-14 reductase to heterocyclic amine antifungals
Source: NPJ Antimicrob Resist. 2025 Oct 1;3:83. doi: 10.1038/s44259-025-00155-7 (PMC12489058; doi:10.1038/s44259-025-00155-7)
Supplement: Supplementary file 1 — Arnold & Chartrain ERG24, Supplementary material [file 44259_2025_155_MOESM1_ESM.pdf]

Resistance of ERG24 sterol C-14 reductase to heterocyclic amine antifungals

Corinne J. Arnold[*#], Laetitia Chartrain[*], David M. Lawson and James K.M. Brown[‡]

John Innes Centre, Norwich Research Park, Colney, Norwich, NR4 7UH, U.K.

[*] These authors made equal contributions to this paper.

[¶] Present address of C.J. Arnold: Camena Bioscience, Chesterford Research Park, Cambridge, CB10 1XL, U.K.

[‡] Author for correspondence: james.brown@jic.ac.uk. ORCID: 0000-0001-8719-2268.

Supplementary figures and tables

Supplementary Information: Table of Contents

Supplementary Figure 1. Outline of the ergosterol synthesis pathway from squalene onwards in *Saccharomyces cerevisiae* and most other fungi, with sterols and precursors, enzymes and antifungals.

Supplementary Figure 2. Structures of sterols relevant to this paper.

Supplementary Figure 3. Multiple sequence alignment of ERG24 enzymes from *Blumeria graminis* f.sp. *tritici* JIW11, *Blumeria graminis* f.sp. *hordei* DH14, *Saccharomyces cerevisiae* S288C and *Methylotuvimicrobium alcaliphilum*.

Supplementary Figure 4. Dose-responses to amorolfine of all three V295L base-edited strains of yeast strain S288C.

Supplementary Figure 5. Dose-response curves of base-edited strains of yeast strain S288C showing variation in responses to the sterol synthesis inhibitor antifungals terbinafine, an inhibitor of ERG1, and ketoconazole, an inhibitor of ERG11 (CYP51).

Supplementary Figure 6. *S. cerevisiae* ERG24 structure predicted using AlphaFold2, with per residue pLDDT plots, a PAE plot and a structural overview coloured according to pLDDT score.

Supplementary Figure 7. *Blumeria graminis* f.sp. *tritici* ERG24 structure predicted using AlphaFold2.

Supplementary Figure 8. *Blumeria graminis* f.sp. *hordei* ERG24 structure predicted using AlphaFold2.

Supplementary Figure 9. Comparison of ERG24 structures with a superposition of the top ranked AlphaFold2 models for *S. cerevisiae*, *B. graminis* f.sp. *tritici* and *B. graminis* f.sp.*hordei*, and the crystal structure from *M. alcaliphilum*, which are shown in cartoon representation, with a detailed view of key residues and the NADPH cofactor.

Supplementary Figure 10. Predictions of antifungals' docking to ERG24 in *S. cerevisiae* with both enantiomers of fenpropimorph, amorolfine and fenpropidin and the achiral tridemorph.

Supplementary Table 1. Isolates of *Blumeria graminis* f.sp. *tritici* and f.sp. *hordei* used in this study, with median effective doses (ED50) of Corbel (active ingredient fenpropimorph 750 g/L).

Supplementary Table 2. Statistical analysis of ED50s of *Blumeria graminis* f.sp. *tritici* isolates. (a) Glasshouse isolates, in relation to the number of sprays of Corbel applied during the period of sampling. (b) Comparison of glasshouse isolates with samples from the air spora above fields.

Supplementary Table 3. Residues in ERG24 in fungi which are predicted to be critical for enzyme function and interactions with phenylpropyl cyclic amine antifungals, including homology of ERG24 in *Blumeria graminis* f.sp. *tritici* and f.sp. *hordei*, *Saccharomyces cerevisiae* and SR1 in the bacterium *Methylotuvimicrobium alcaliphilum*.

Supplementary Table 4. Distribution of amino-acids at sites homologous to residues 291 and 295 in ERG24 of *Blumeria graminis* throughout the tree of life.

Supplementary Table 5. Parameters of dose-response curves of *Saccharomyces cerevisiae* S288C strains to sterol synthesis inhibitor antifungals, measured as fungal growth.

Supplementary Table 6. Growth parameters of *Saccharomyces cerevisiae* S288C and base-edited strains in the absence of an antifungal.

Supplementary Table 7. Sequences of primers designed to amplify and sequence the *Erg24* gene in *Blumeria graminis* f.sp. tritici (*Bgt*) and f.sp. *hordei* (*Bgh*) and *Erg2* in *Bgt*. F indicates a forward primer, R indicate a reverse primer.

Supplementary Table 8. Primers used in *Saccharomyces cerevisiae* strain S288C to test the effect of mutations detected in *Blumeria graminis* ERG24 on responses to cyclic amine antifungals. (A) Primers used in base editing to introduce single-nucleotide substitutions. (B) Primers used to check that mutations had been introduced correctly.

Supplementary Table 9. Identification of residues which differ between host and fungal pathogen taxa in charge, polarity or size in the linker between transmembrane domains 6 and 7 of ERG24. (A) Major taxa of crop plants and major taxa of fungi pathogens of crops. (B) Humans, domesticated animals and important pathogenic fungi.

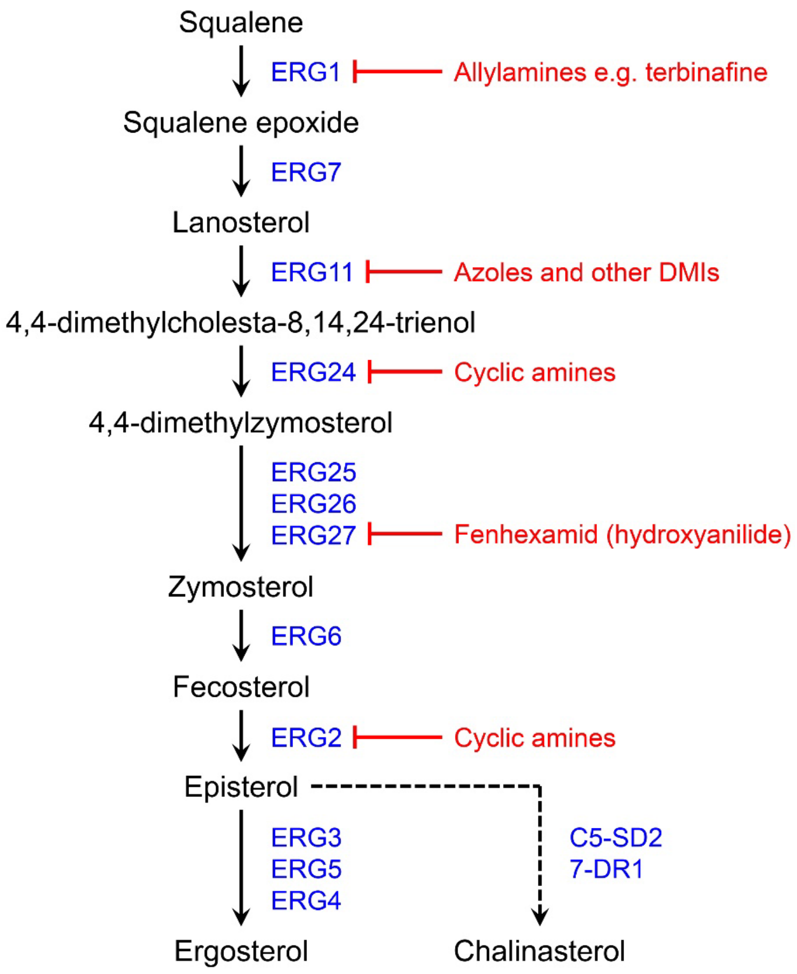

Supplementary Figure 1. Solid line: outline of the ergosterol synthesis pathway from squalene onwards in *Saccharomyces cerevisiae* and most other Dikarya fungi (ascomycetes and basidiomycetes). Dashed line: the main membrane sterol of powdery mildews (Erysiphales), including *Blumeria graminis*, is chalinasterol (24-methylenecholesterol), not ergosterol. Black: sterols and precursors. Blue: enzymes. Red: antifungals discussed in this paper (DMI: sterol C14 demethylation inhibitor).

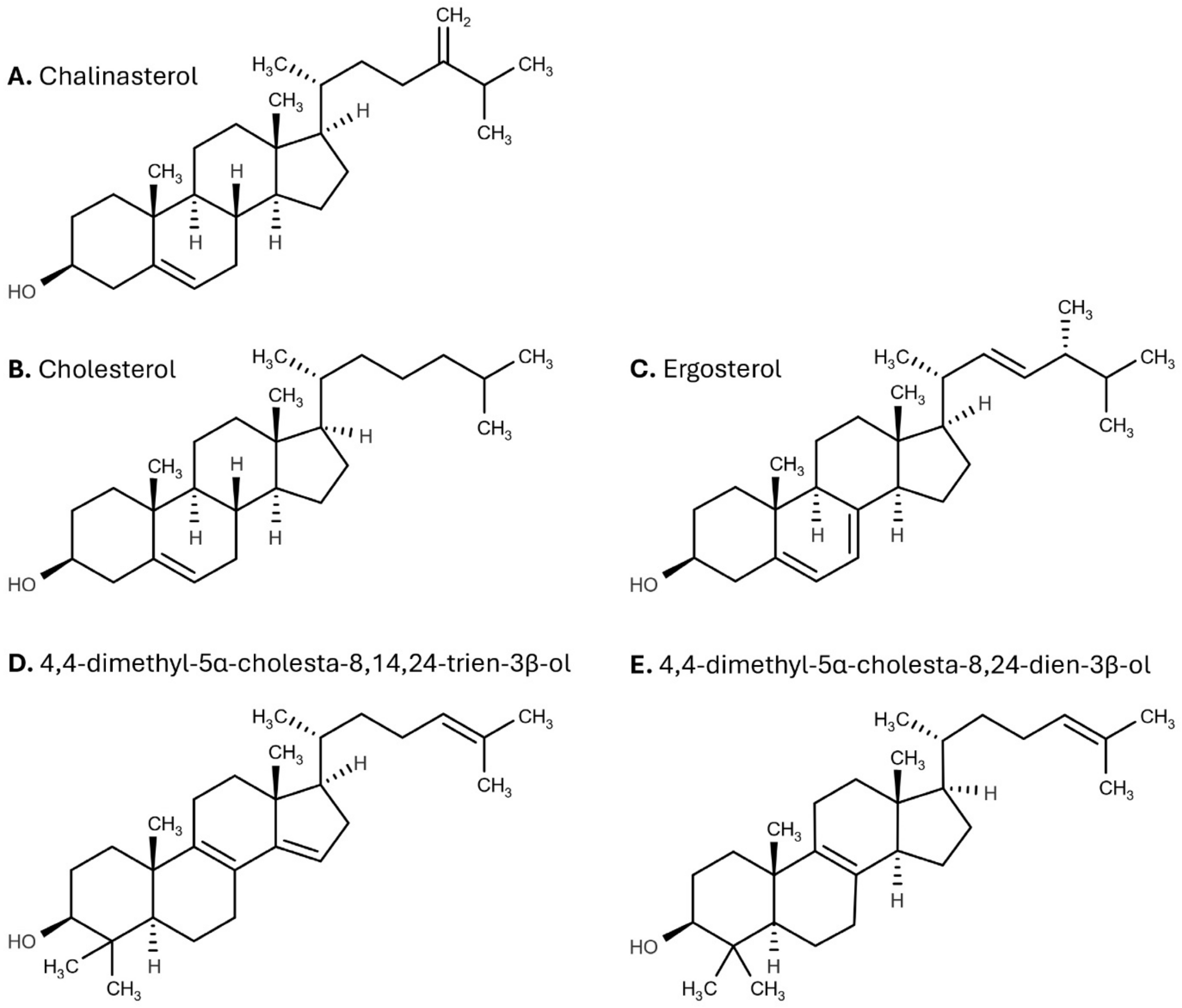

Supplementary Figure 2. Structures of sterols relevant to this paper. **A.** Chalinasterol (ostreasterol, 24-methylene-cholesterol, ergosta-5,24(24^1)-dien-3β-ol), an unusual compound which is the principal membrane sterol of powdery mildew fungi, including *Blumeria graminis*. For comparison, **B.** cholesterol and **C.** ergosterol, the principal membrane sterol of most other Dikarya (ascomycete and basidiomycete fungi). **D.** 4,4-dimethyl-5α-cholesta-8,14,24-trien-3β-ol and **E.** 14-methyllanosterol (4,4-dimethyl-5α-cholesta-8,24-dien-3β-ol), the substrate and product respectively of Δ14-sterol reduction catalysed by ERG24 in *Saccharomyces cerevisiae*. Structures taken from ChEBI.

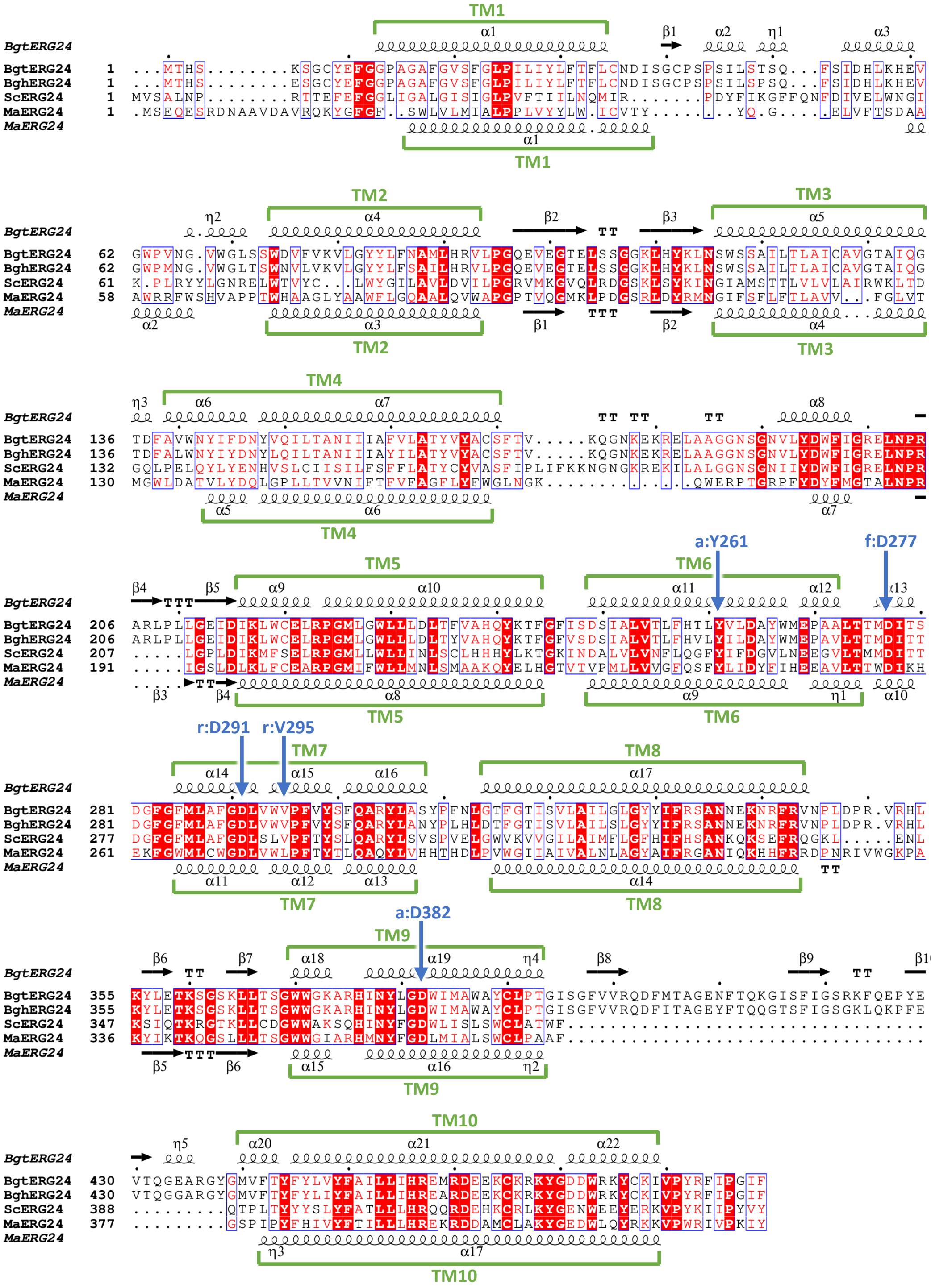

Supplementary Figure 3. Multiple sequence alignment of selected ERG24 enzymes. Sequences of enzymes from *Blumeria graminis* f.sp. *tritici* JIW11 (*Bgt*ERG24), *Blumeria graminis* f.sp. *hordei* DH14 (*Bgh*ERG24), *Saccharomyces cerevisiae* S288C (*Sc*ERG24) and *Methylotuvimicrobium alcaliphilum* (MaERG24) were aligned using Clustal Omega v1.2.4 then displayed using ESPript3.0 (http://espript.ibcp.fr/ESPript/ESPript/). Strictly conserved residues are highlighted with red shaded boxes, and semi-conserved residues are coloured red. Secondary structure elements for BgtERG24 (AlphaFold2 model) and MaERG24 (PDB code 4QUV) are shown above and below the alignment, respectively, where α = α helix, β = β strand, η = 3_{10} helix, TT = β turn. Also labelled in green are the transmembrane segments (TM1-TM10) each of which consists of one to three α-helices, and key residues are indicated in blue with BgtERG24 numbering. The residue labels are preceded by "a" for "active site residue", "r" for "resistance locus" and "f" for "fungicide binding site", with the latter being the most frequently observed interaction site for the heterocyclic nitrogen of all fungicides in the docking simulations.

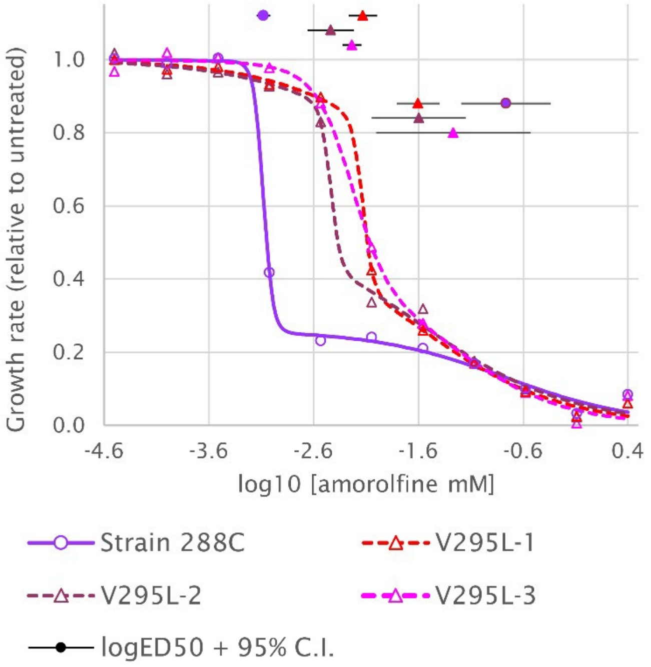

Supplementary Figure 4. Dose-responses to amorolfine of all three V295L base-edited strains of yeast strain S288C. This figure is supplementary to Figure 2A. Construction of yeast strains with amino-acid substitutions introduced by single-nucleotide substitution using CRISPR/Cas9, the test method and curve-fitting procedures are described in Materials & Methods. Median effective doses (ED50) are shown together with 95% confidence intervals.

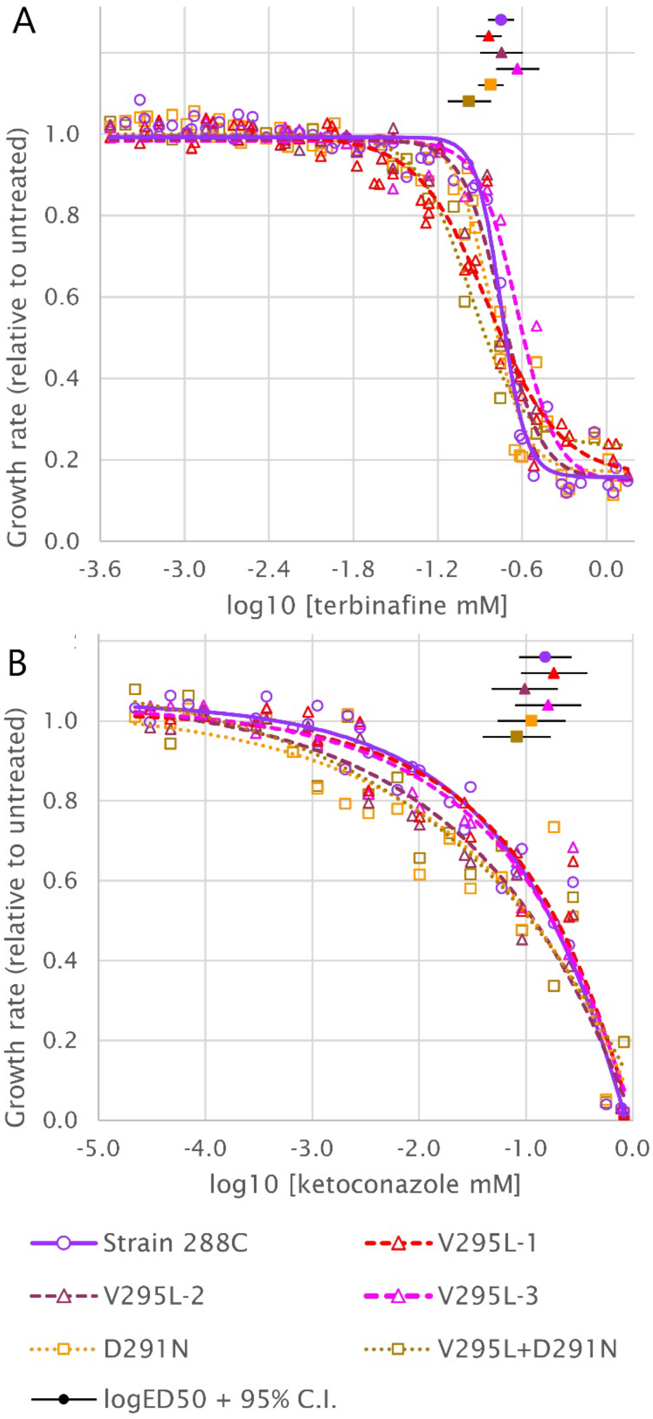

Supplementary Figure 5. Dose-response curves of yeast strain S288C with amino-acid substitutions introduced by single-nucleotide substitution using CRISPR/Cas9, showing variation in responses to sterol synthesis inhibitor antifungals: (A) terbinafine, an inhibitor of ERG1, (B) ketoconazole, an inhibitor of ERG11 (CYP51). Construction of yeast strains, the test method and curve-fitting procedures are described in Materials & Methods. Median effective doses (ED50) are shown together with 95% confidence intervals.

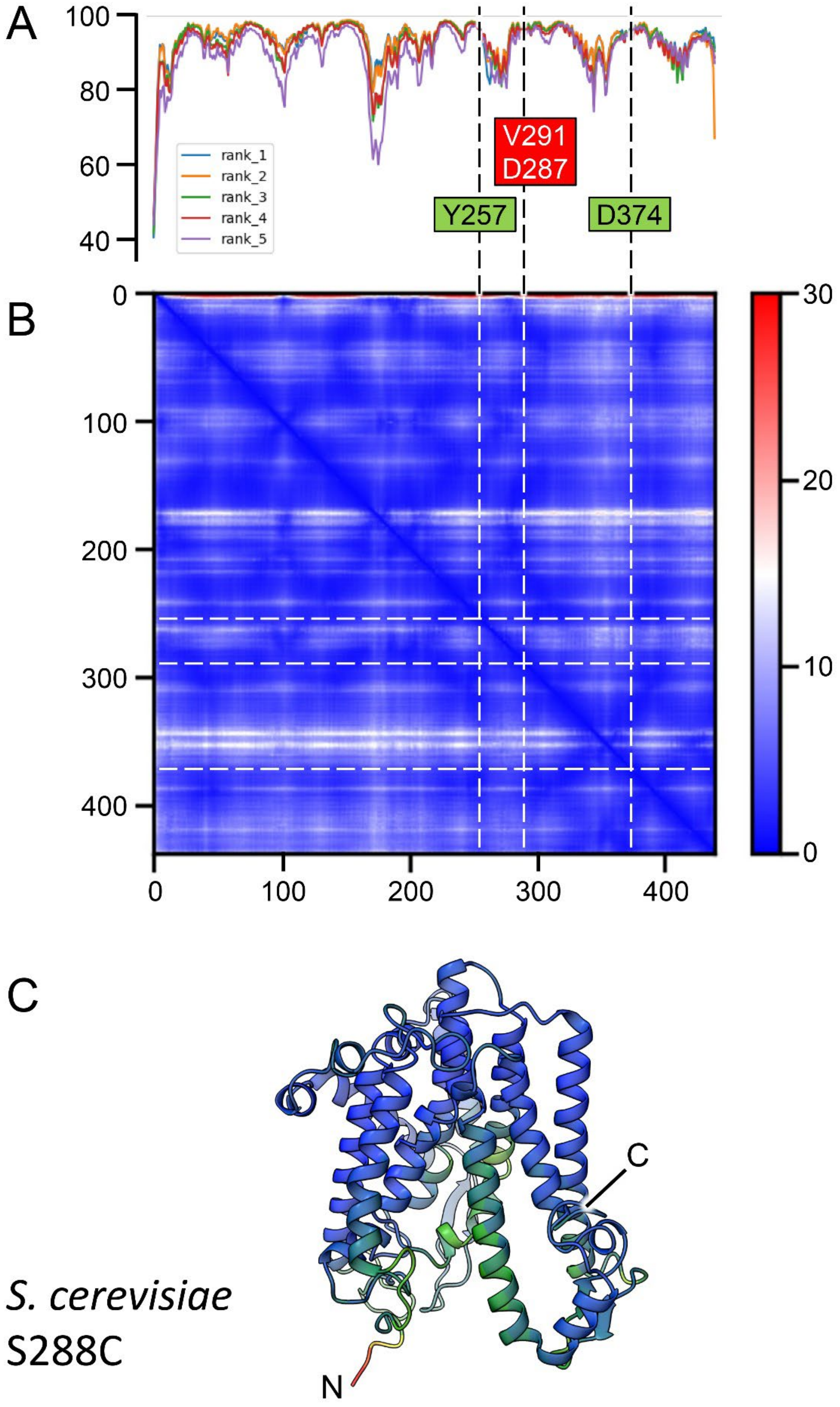

Supplementary Figure 6. *S. cerevisiae* ERG24 structure predicted using AlphaFold2. (A) Per residue pLDDT plots for the five independent models, (B) PAE plot for the rank 1 model, (C) Structural overview of the rank 1 model in cartoon representation coloured according to pLDDT score where residues with values less than 50 are coloured red; residues with values in the range 50-100 are shown in rainbow colouration from red through to blue. The dashed lines plotted onto panels A and B indicate the positions of predicted catalytic residues (black text on green labels) and mutation sites (white text on red labels). In all cases, these residues lie in regions with high pLDDT scores, and the dashed lines intersect at points within dark blue regions of the PAE plot, indicative of high confidence in the relative placement of these residues.

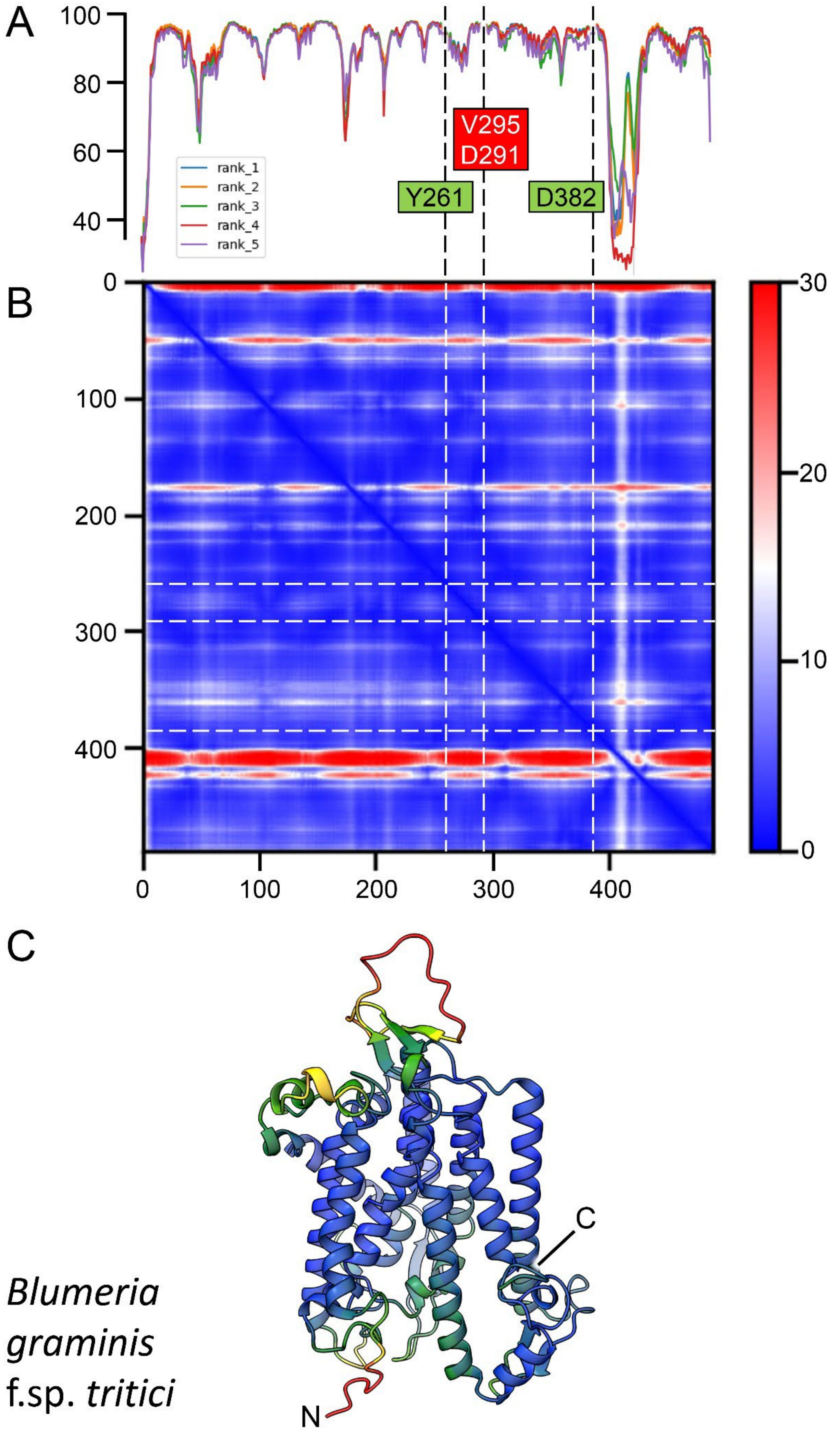

Supplementary Figure 7. *Blumeria graminis* f.sp. *tritici* ERG24 structure predicted using AlphaFold2. Panels (A), (B) and (C) and details of labelling as in Supplementary Figure 6.

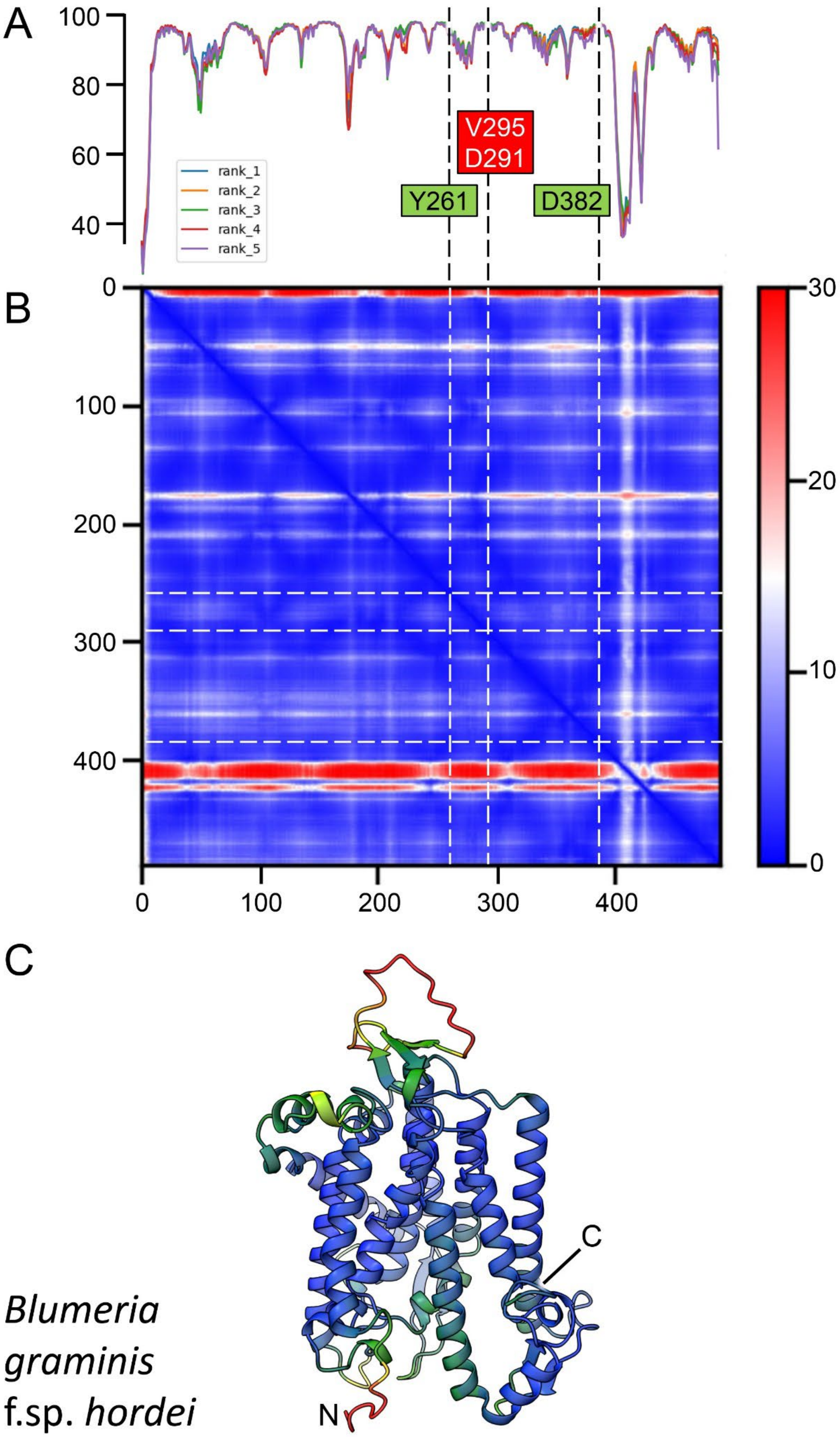

Supplementary Figure 8. *Blumeria graminis* f.sp. *hordei* ERG24 structure predicted using AlphaFold2. Panels (A), (B) and (C) and details of labelling as in Supplementary Figure 6.

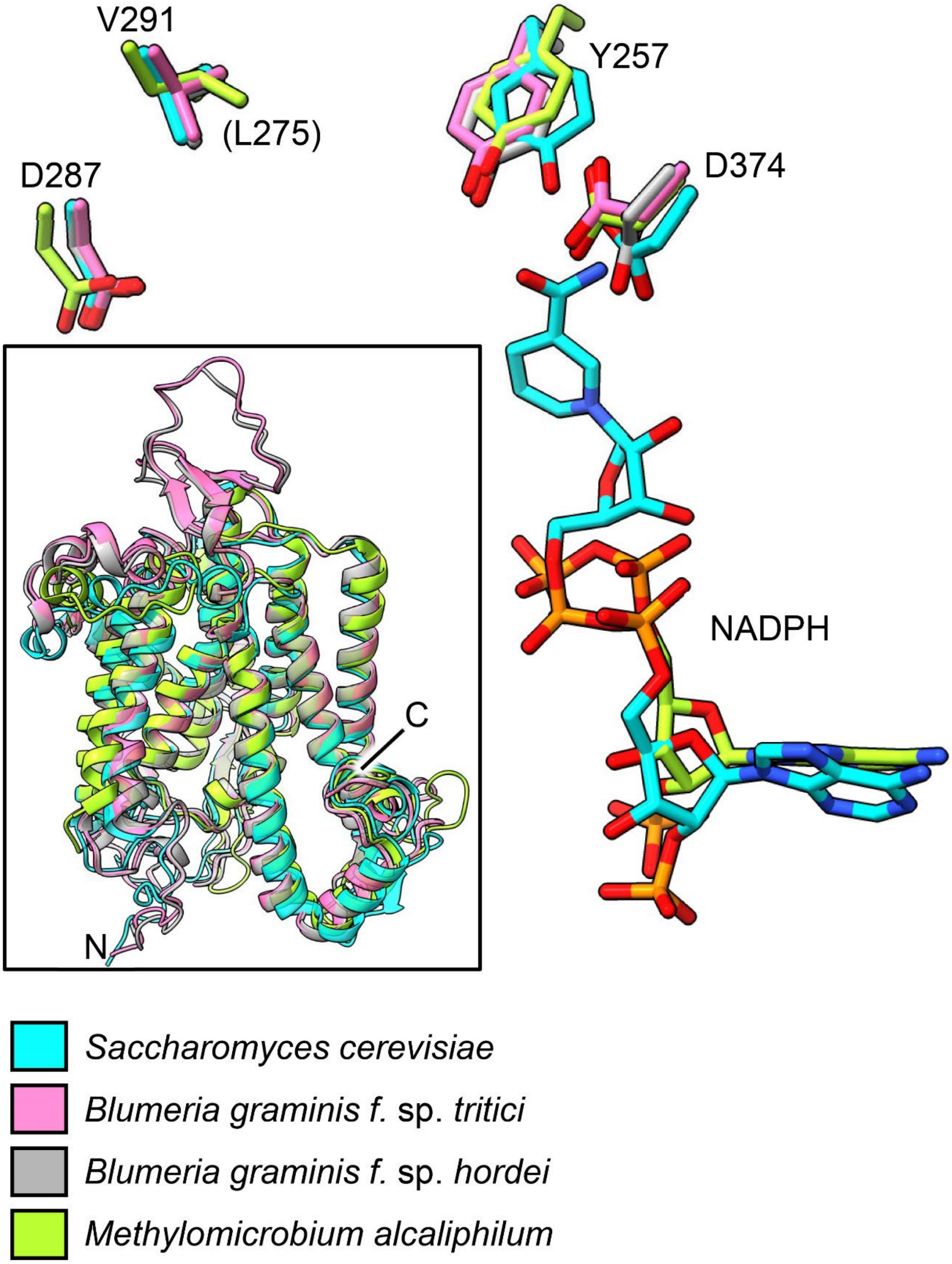

Supplementary Figure 9. Comparison of ERG24 structures. The inset show a superposition of the top ranked AlphaFold2 models for *S. cerevisiae*, *B. graminis* f.sp. *tritici* and *B. graminis* f.sp. *hordei*, and the crystal structure from *M. alcaliphilum*, which are shown in cartoon representation. The remainder of the figure shows just the key residues from this superposition. Also shown is the NADPH cofactor docked into the *S. cerevisiae* model, and the partial cofactor observed in the *M. alcaliphilum* crystal structure. The numbering is with respect to the *S. cerevisiae* sequence apart from for the non-conserved Leu275 in *M. alcaliphilum*, which is shown in brackets. The 3D structures are displayed using ChimeraX v1.8[80].

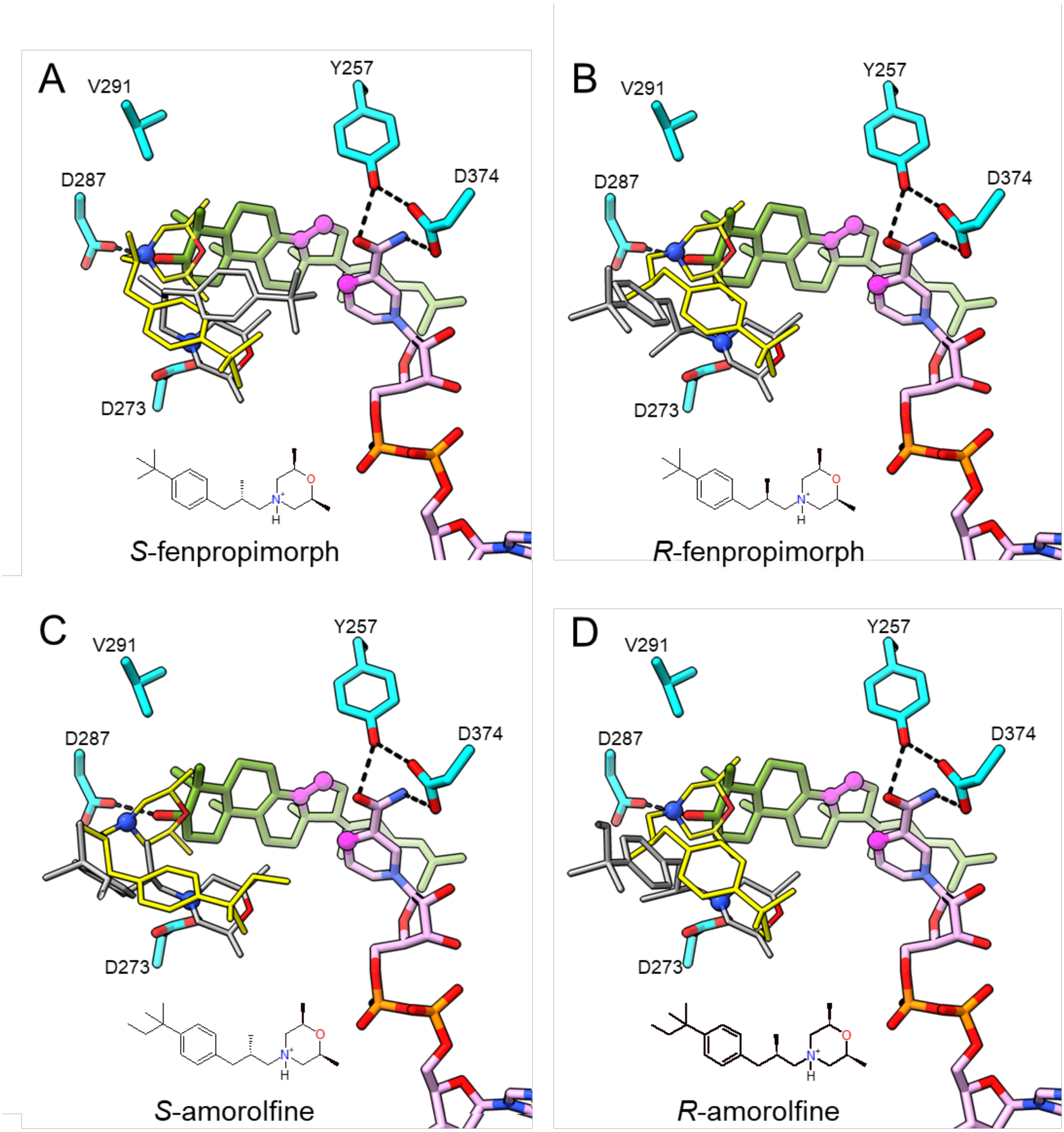
A
V291
Y257
D287
D374
D273
S-fenpropimorph
B
V291
Y257
D287
D374
D273
R-fenpropimorph
C
V291
Y257
D287
D374
D273
S-amorolfine
D
V291
Y257
D287
D374
D273
R-amorolfine

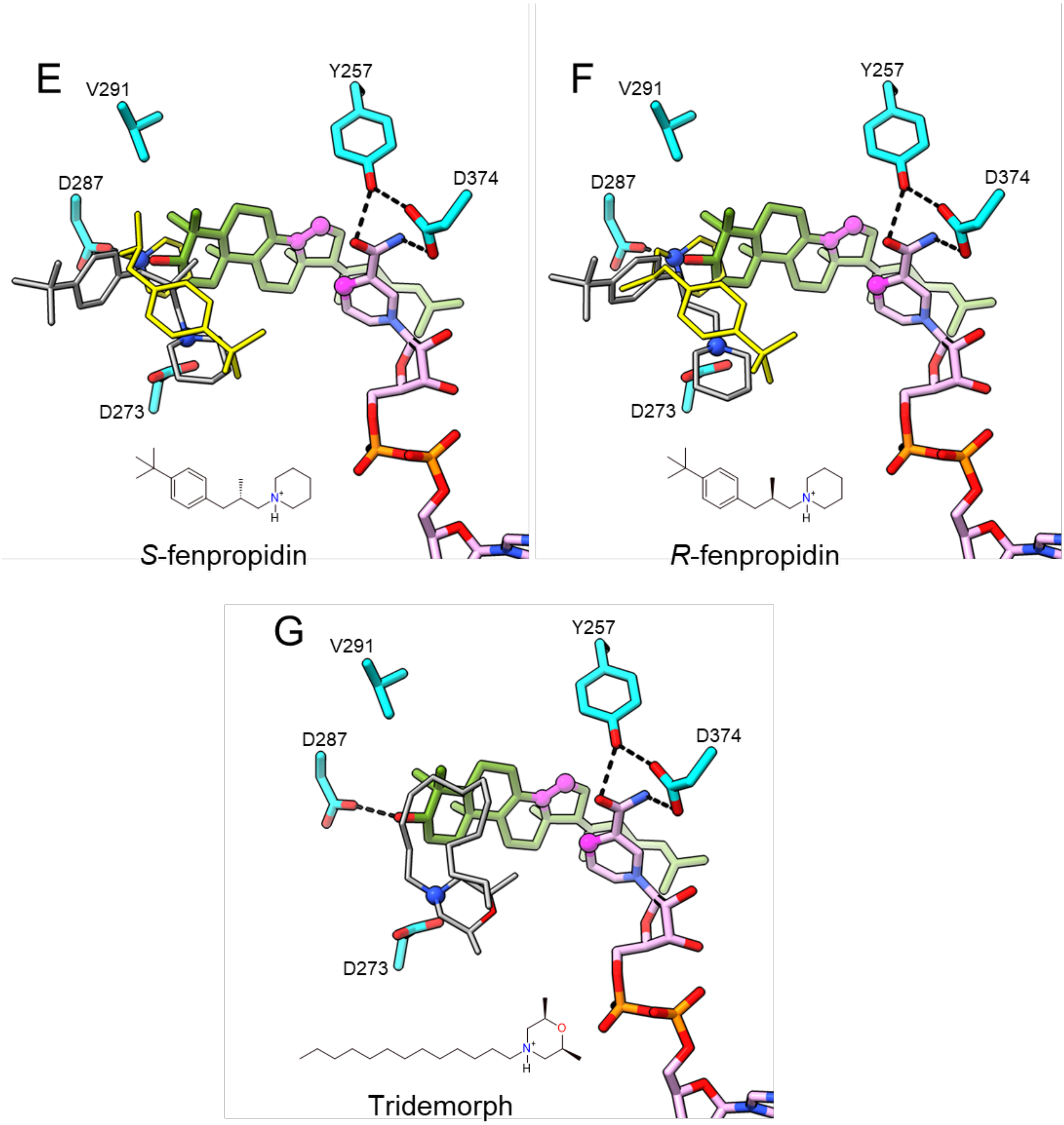

Supplementary Figure 10. Predictions of antifungals' docking to ERG24 in *S. cerevisiae*. Comparisons of docking simulations using both enantiomers of the antifungals (A, B) fenpropimorph, (C,D) amorolfine, (E,F) fenpropidin and (G) the achiral tridemorph. Shown is the same close-up view as used in Fig. 3C with key residues and ligands depicted in stick representation as follows: predicted catalytic residues Tyr257 and Asp374, and resistance-conferring mutation sites Val291 and Asp287, and the conserved Asp273 that may play a role in antifungal binding (cyan carbons); docked cofactor (pale pink carbons); docked substrate (green carbons). For clarity, only two of the nine poses are shown from each docking simulation, performed in the absence of cofactor and substrate although both are displayed for reference. One pose is shown where the heterocyclic nitrogen (highlighted as a blue sphere) is poised to hydrogen bond to Asp287 (thin sticks; yellow carbons) and, in the other pose, the nitrogen is poised to hydrogen bond to Asp273 (thin sticks; grey carbons). None of the poses for tridemorph interacted with Asp287, so only a single pose is shown in panel G. For each panel, a 2D representation of the corresponding fungicide structure is given as an inset. The 3D structures are displayed using ChimeraX v1.8[80] and the 2D sketches of the fungicides were produced using ACD/ChemSketch 2022.2.3.

Supplementary Table 1. Isolates of *Blumeria graminis* f.sp. *tritici* and f.sp. *hordei* used in this study, with median effective doses (ED50) of Corbel (active ingredient fenpropimorph 750 g/L). ED50 were estimated as described in the Material & Methods.

Isolate	Corbel ED50 (with 95% confidence interval)	ERG24 residue 295	ERG2 sequenced
**a. *Blumeria graminis* f.sp. *tritici* (wheat powdery mildew)**			
JIC glasshouses, 2014			
CAW14S6104	99.9 (51.9 - 192)		
CAW14S6303	80.3 (56.8 - 113)	L	
CAW14S6306	73.1 (45.2 - 118)		
CAW14S6307	118 (81.9 - 171)	L	✓
CAW14S6312	178 (87.9 - 359)		
CAW14S6317	118 (82.6 - 168)	L	
CAW14S6318	98.2 (67.7 - 143)	L	✓
JIC glasshouses, 2015			
• *No spray of Corbel in 2015*			
CAW15S6320	122 (85.6 - 173)	L	
CAW15S6325	57.6 (35.2 - 94.3)		
CAW15S6328	230 (110 - 479)		
CAW15S6330	187 (130 - 270)	L	
CAW15S6332	197 (136 - 285)	L	✓
CAW15S6334	273 (165 - 452)		
• *1 spray of Corbel in 2015 before sampling*			
CAW15S6335	208 (146 - 297)	L	
CAW15S6336	167 (119 - 234)	L	
CAW15S6337	180 (126 - 256)	L	✓
CAW15S6338	232 (158 - 341)	L	
CAW15W101	126 (89.3 - 179)		
CAW15W102	199 (139 - 286)	L	✓
CAW15W103	180 (120 - 270)	L	✓
CAW15W104	147 (102 - 212)		
CAW15W105	88 (60.7 - 128)		
• *2 sprays of Corbel in 2015 before sampling*			
CAW15S6339	130 (91.4 - 185)	L	
CAW15S6340	126 (90.3 - 176)		
CAW15S6341	168 (114 - 249)		
CAW15S6342	214 (152 - 300)	L	
CAW15S6343	275 (192 - 395)	L	
Air spora above fields in Norfolk, England, 2015			
ADW1503	153 (95.1 - 248)		
ADW1506	182 (112 - 294)		
AEW1503	380 (228 - 632)		
AEW1504	60.3 (42.5 - 85.5)	L	
ASW1502	155 (90.2 - 268)		
ASW1504	402 (245 - 658)		
CMW1502	84.6 (55.7 - 129)	L	
CMW1505	191 (112 - 325)		
CMW1506	75.9 (45.8 - 126)		
EOW1501	112 (79 - 159)	L	
JBW1503	262 (124 - 552)		
JBW1504	159 (110 - 231)	L	

TKW1503	70.9 (49.3 - 102)	L	
TKW1504	124 (84.5 - 183)		
TKW1506	204 (130 - 320)		
Reference isolates			
JIW11 (UK 1985)	9.19 (6.4 - 13.2)	V	✓
GAP.B.2.2 (USA 2013)	12.2 (8.6 - 17.3)	V	✓
OKH.A.2.1 (USA 2013)	68.8 (45.6 - 104)	V	✓
OKS(14)A.2.3 (USA 2014)	9.19 (6.35 - 13.3)	V	✓
94202 (CH 1994)	10.7 (7.53 - 15.1)	V	
96224 (CH 1996)	10.9 (7.58 - 15.8)	L	✓

b. *Blumeria graminis* f.sp. *hordei* (barley powdery mildew)

Isolate	Corbel ED50 (with 95% confidence interval)	ERG24 residues	
		291	295
CC52 (UK 1985)	Sensitive	D	V
DH14 (UK1986)	Sensitive	D	V
W4 (Norfolk 2017)	10.7 (8.38 – 13.8)	D	L
CC148 (UK 1990)	29.8 (23.4 – 38.1)	N	V

Supplementary Table 2. Statistical analysis of ED50s of *Blumeria graminis* f.sp. *tritici* isolates.

a. Median effective dose (ED50, ml ha^{-1}) of Corbel (750 g L^{-1} fenpropimorph) for *Blumeria graminis* f.sp. *tritici* isolates from John Innes Centre glasshouses in 2014 and 2015, in relation to the number of sprays (0, 1 or 2) of Corbel applied to wheat between the first and last set of isolates being sampled. Isolates were tested in four batches and ED50 estimated as described in Materials & Methods.

Response variate: $\log_{10}$(ED50), weighted by inverse of squared standard error

Linear mixed model

Variance components (with standard error) of random effect

Term	VC	SE
Batch of tests	0.069	0.058
Isolate	0.015	0.007
Residual	3.141	0.754

Analysis of variance of fixed effect

Term	Wald statistic	Numerator DF	Denominator DF	F-test probability
Sprays	2.00	1	23.4	0.2

Model parameters

Term	Coefficient	SE
Constant	2.162	0.135
Sprays	0.055	0.039

Model formula

$\log_{10}$ED50 = 2.16 + 0.055 x Sprays

ED50 = 145 x (Sprays ^ 1.14)

b. Comparison of Corbel ED50 of *Bgt* isolates sampled from JIC glasshouses in 2014 and 2015 and from the air spora above Norfolk fields in 2015.

Response variate: $\log_{10}$(ED50), weighted by inverse SE

Linear mixed model

Variance components (with standard error) of random effects

Term	VC	SE
Batch of tests	0.081	0.068
Isolate	0.018	0.007
Residual	3.401	0.662

Analysis of variance of fixed effect

Term	Wald	NDF	DDF	P (F test)
Group	8.07	2	36.2	0.03

Predicted means

Term	Mean	SE
JIC 2014	1.999	0.158
JIC 2015	2.207	0.147
Field 2015	2.113	0.151

Comparison of means (Fisher's least significant difference)

Comparison	Difference	SED	P (t-test)
JIC 2014 – 2015	−0.209	0.076	0.01
Field – JIC 2014	0.115	0.084	0.2
Field – JIC 2015	−0.094	0.063	0.1

Supplementary Table 3. Residues in ERG24 in fungi which are predicted to be critical for enzyme function and interactions with phenylpropyl cyclic amine antifungals, and homology with SR1 in the bacterium *Methylotuvimicrobium alcaliphilum*.

**Residue**			
***Blumeria graminis* f.sp. *tritici* JIW11 & f.sp. *hordei* DH14**	***Saccharomyces cerevisiae* S288C**	***Methylotuvi-microbium alcaliphilum***	**Proposed role**
Tyr261	Tyr 257	Tyr241	Catalytic: protonation of substrate at C15
Asp277	Asp273	Asp257	Invariant residue: predicted to bind to heterocycle N of antifungals in most simulations
Asp291	Asp287	Asp271	Involved in resistance: D291N in *Bg* & D287N in *Sc* increases the lower ED50 of phenylpropylamines but reduces the ED50 of tridemorph; may be involved in sterol binding and possible direct interaction with antifungals
Val295	Val291	Leu275	Involved in resistance: V295L in *Bg* (V291L in *Sc*) increases or abolishes the lower ED50 of phenylpropylamines; adjacent to sterol binding site but no direct interaction with antifungals in simulations
Asp382	Asp374	Asp363	Catalytic: coordinates carbamoyl group of cofactor nicotinamide moiety and hydroxyl of Tyr261; may polarise the latter facilitating substrate protonation
Arg457	Arg406	Arg395	Cofactor binding: interaction with diphosphate group

Supplementary Table 4. Distribution of amino-acids at sites homologous to residues 291 and 295 in ERG24 of *Blumeria graminis* throughout the tree of life. Complete sequences of ERG24 homologues are in 'Arnold & Chartrain, ERG24 Supplementary Data.xlsx'.

<table>
<tr><th colspan="9" rowspan="2">Outline classification[1]</th><th rowspan="2">Principal membrane sterols</th><th rowspan="2">Representative species</th><th rowspan="2">Description</th><th rowspan="2">Sequence ID</th><th colspan="2">Residue</th></tr>
<tr><th>Bgt291</th><th>Bgt295</th></tr>
<tr><td rowspan="15">Amorphea</td><td rowspan="14">Obazoa</td><td rowspan="13">Opisthokonta</td><td rowspan="11">Eumycota</td><td rowspan="7">Symbiomycota</td><td rowspan="6">Dikarya</td><td rowspan="4">Ascomycota</td><td rowspan="3">Saccharo-myceta</td><td rowspan="2">Pezizomycotina</td><td>Chalinasterol</td><td>Blumeria graminis f.sp. tritici</td><td>Wheat powdery mildew</td><td>PQ848564</td><td>D</td><td>V</td></tr>
<tr><td>Chalinasterol</td><td>Blumeria graminis f.sp. hordei</td><td>Barley powdery mildew</td><td>PQ848567</td><td>D</td><td>V</td></tr>
<tr><td>Saccharomycotina</td><td>Ergosterol</td><td>Saccharomyces cerevisiae</td><td>Brewer's yeast</td><td>NP_014119.1</td><td>D</td><td>V</td></tr>
<tr><td colspan="2">Taphrinomycotina</td><td>Ergosterol</td><td>Schizosaccharomyces pombe</td><td>Fission yeast</td><td>NP_596767.1</td><td>D</td><td>V</td></tr>
<tr><td rowspan="2">Basidio-mycota</td><td colspan="2">Orthomycotina</td><td>Ergosterol</td><td>Psilocybe cubensis</td><td>Magic mushroom</td><td>XP_047749130.1</td><td>D</td><td>V</td></tr>
<tr><td colspan="2">Pucciniomycotina</td><td>Stigmasta-7,24(28)-dienol
Stigmast-7-enol</td><td>Puccinia graminis f.sp. tritici</td><td>Wheat stem rust</td><td>KAH9464627.1</td><td>D</td><td>V</td></tr>
<tr><td colspan="4">Glomeromycota</td><td>24-Ethylcholesterol</td><td>Rhizophagus irregularis</td><td>Arbuscular mycorrhiza</td><td>EXX72918.1</td><td>N</td><td>L</td></tr>
<tr><td colspan="5">Mucoromycota</td><td>Ergosterol
22,23-Dihydroergosterol</td><td>Mucor lusitanicus</td><td>Mucormycosis, "black fungus"</td><td>OAD00002.1</td><td>N</td><td>V</td></tr>
<tr><td colspan="5">Entomophthoromycota</td><td>24-Methylcholesterol</td><td>Entomophthora muscae</td><td>Parasite of flies</td><td>KAJ9086470.1</td><td>D</td><td>V</td></tr>
<tr><td colspan="5">Blastocladiomycota</td><td>Cholesterol</td><td>Allomyces javanicus</td><td>Zoosporic fungus</td><td>KAJ3360781.1</td><td>D</td><td>V</td></tr>
<tr><td colspan="5">Chytridiomycota</td><td>Cholesterol</td><td>Hyaloraphidium curvatum</td><td>Chytrid</td><td>KAI9027873.1</td><td>D</td><td>V</td></tr>
<tr><td colspan="3" rowspan="2">Animalia</td><td colspan="3">Protostomia</td><td>Cholesterol</td><td>Octopus sinensis</td><td>East Asian octopus</td><td>XP_029642509.1</td><td>D</td><td>V</td></tr>
<tr><td colspan="3">Deuterostomia</td><td>Cholesterol</td><td>Homo sapiens</td><td>Human</td><td>NP_003264.2</td><td>D</td><td>V</td></tr>
<tr><td colspan="7">Apusomonadida</td><td>Unknown</td><td>Thecamonas trahens</td><td>Marine protist</td><td>XP_013762997.1</td><td>D</td><td>V</td></tr>
<tr><td colspan="8">Amoebozoa</td><td>Dictyosterol
24-Dihydrodictyosterol</td><td>Dictyostelium discoideum</td><td>Slime mould</td><td>XP_638613.1</td><td>D</td><td>V</td></tr>
<tr><td rowspan="4">Bikonta</td><td colspan="2" rowspan="3">Diapho-retickes</td><td colspan="3" rowspan="2">Archae-plastida</td><td colspan="3">Viridiplantae</td><td>Sitosterol,
Campesterol, etc</td><td>Triticum aestivum</td><td>Wheat</td><td>XP_044423045.1</td><td>D</td><td>I</td></tr>
<tr><td colspan="3">Rhodophyta</td><td>Cholesterol</td><td>Galdieria sulphuraria</td><td>Red alga</td><td>XP_005707403.1</td><td>D</td><td>I</td></tr>
<tr><td colspan="6">SAR, Phaeophyceae</td><td>Fucosterol</td><td>Ectocarpus siliculosus</td><td>Brown alga</td><td>CBJ27514.1</td><td>D</td><td>L</td></tr>
<tr><td colspan="8">Euglenozoa</td><td>Ergosterol</td><td>Leishmania tarentolae</td><td>Parasitic protist</td><td>GET91576.1</td><td>D</td><td>V</td></tr>
<tr><td colspan="9">Bacteria, Methylococcaceae</td><td>4-Methyl-Δ^8-sterols</td><td>Methylotuvimicrobium alcaliphilum</td><td>Methanotroph</td><td>WP_014147797.1</td><td>D</td><td>L</td></tr>
</table>

[1]Protein sequences were annotated as sterol Δ14 reductase in the NCBI database. In general, the sequence chosen to represent a taxon was the one most homologous to that of *Blumeria graminis* f.sp. *tritici* (*Bgt*), unless a much better-studied species had slightly lower homology. Protein sequences were aligned by MUSCLE 3.8.425 on the EMBL-EBI server. Proteins annotated as ERG4/ERG24 sterol reductases were not included, nor were high-level taxa in which no sterol Δ14-reductase was detected by homology with *Bgt*, *O. sinensis* or *T. aestivum*.

Supplementary Table 5. Parameters of dose-response curves of *Saccharomyces cerevisiae* S288C strains to sterol synthesis inhibitor antifungals, measured as fungal growth (with standard errors).

In all models: g = fungal growth; L = log(dose); m: L at point of inflection; b: gradient parameter; c: asymptotic value of g.

Amorolfine (Figure 2A and Supplementary Figure 4)

Double logistic model: $g = (1 - c)[1 + \exp\{-b_1(L - m_1)\}]^{-1} + c[1 + \exp\{-b_2(L - m_2)\}]^{-1}$

lower ED50 = m_1 & upper ED50 = m_2

Strain	Figure	m_1	b_1	c	m_2	b_2
S288C	2B	−2.68 (0.01)	−12.62 (0.88)	0.27 (0.02)	−0.77 (0.09)	−1.81 (0.22)
	S5B	−3.08 (0.03)	−22.16 (13.15)	0.26 (0.05)	−0.77 (0.21)	−1.57 (0.48)
S288C-V291V	2B	−2.67 (0.01)	−15.28 (1.87)	0.31 (0.02)	−0.79 (0.07)	−2.23 (0.27)
S288C-rev	2B	−2.67 (0.01)	−15.00 (1.68)	0.32 (0.02)	−0.81 (0.05)	−2.64 (0.29)
S288C-V291L-1	2B	−1.95 (0.01)	−10.86 (1.27)	0.35 (0.06)	−0.95 (0.12)	−2.29 (0.28)
	S5B	−2.13 (0.07)	−20.23 (16.24)	0.54 (0.09)	−1.60 (0.10)	−1.49 (0.16)
S288C-V291L-2	S5B	−2.44 (0.11)	−21.00 (23.13)	0.56 (0.14)	−1.60 (0.22)	−1.40 (0.18)
S288C-V291L-3	S5B	−2.23 (0.05)	−5.89 (1.39)	0.42 (0.23)	−1.27 (0.37)	−1.88 (0.42)
S288C-D287N	2B	−2.16 (0.01)	−12.20 (1.55)	0.32 (0.06)	−0.99 (0.15)	−2.04 (0.32)
S288C-double	2B	−2.33 (0.01)	−9.27 (0.74)	0.25 (0.05)	−1.00 (0.16)	−1.87 (0.29)

Fenpropidin (Figure 2B)

Double Gompertz model (d-Gom): $g = \{c + (1 - c)(\exp[-\exp\{-b_1(L - m_1)\}])\}\exp[-\exp\{-b_2(L - m_2)\}]$

lower ED50 ≈ $m_1 - \log(\log 2)/b_1$ & upper ED50 = $m_2 - \log(\log 2)/b_2$

or Gompertz model (Gom) $g = \exp[-\exp\{-b(L - m)\}]$; ED50 = $m - \log(\log 2)/b$

Strain	Model	Lower ED50	m_1	b_1	c	Upper ED50	m_2	b_2
S288C	d-Gom	0.03 (0.01)	0.04 (0.01)	−42.22	0.45 (0.07)	0.78 (0.06)	0.90 (0.06)	−3.07 (0.42)
-V291V	d-Gom	0.03 (0.02)	0.04 (0.02)	−31.19	0.48 (0.06)	0.74 (0.05)	0.89 (0.05)	−2.39 (0.22)
-rev	d-Gom	0.10 (0.03)	0.16 (0.03)	−6.52	0.55 (0.13)	0.76 (0.07)	0.92 (0.07)	−2.34 (0.37)
-V291L-1	Gom					0.95 (0.01)	1.00 (0.01)	−7.78 (0.36)
-D287N	Gom					0.71 (0.01)	0.78 (0.01)	−5.61 (0.52)
-double	Gom					0.76 (0.01)	0.81 (0.01)	−8.36 (0.83)

Fenpropimorph (Figures 2C and 2D)

Double logistic model (d-log): $g = (1-c)[1+\exp\{-b_1(L-m_1)\}]^{-1} + c[1+\exp\{-b_2(L-m_2)\}]^{-1}$

lower median effective dose (ED50) = m_1 & upper ED50 = m_2

or logistic model (log): $g = (1-c)[1+\exp\{-b(L-m)\}]^{-1} + c$; ED50 = m

Strain	Fig.	Model	m_1	b_1	c	m_2	b_2
S288C	2C	log	−0.70 (0.01)	−7.22 (0.62)	0.28 (0.02)		
	2D	d-log	−1.51 (0.02)	−9.44 (1.68)	0.52 (0.03)	0.08 (0.04)	−4.19 (0.61)
S288C-V291V	2C	log	−0.75 (0.02)	−6.98 (0.72)	0.35 (0.02)		
S288C-L291V	2C	log	−0.77 (0.02)	−9.41 (1.29)	0.29 (0.02)		
S288C-V291L-1	2C	log	0.22 (0.05)	−8.28 (1.88)	0.26 (0.15)		
	2D	log				−0.03 (0.01)	−4.09 (0.19)
S288C-V291L-2	2D	log				−0.01 (0.01)	−4.36 (0.16)
S288C-V291L-3	2D	log				−0.06 (0.01)	−4.00 (0.19)
S288C-D287N	2C	log	0.00 (0.03)	−6.27 (0.66)	0.34 (0.05)		
S288C-double	2C	log	−0.28 (0.02)	−8.24 (1.15)	0.32 (0.03)		

Tridemorph (Figure 2E)

Logistic model: $g = [1+\exp\{-b(L-m)\}]^{-1}$; ED50 = m

Strain	m	b
S288C	1.01 (0.02)	−7.01 (0.75)
S288C-V291V	1.03 (0.01)	−7.48 (0.54)
S288C-rev	1.03 (0.01)	−8.16 (0.67)
S288C-V291L-1	1.13 (0.01)	−13.65 (1.88)
S288C-D287N	0.62 (0.02)	−4.29 (0.33)
S288C-double	0.19 (0.08)	−1.90 (0.19)

Terbinafine (Supplementary Figure 5A)

Logistic model: $g = (1-c)[1+\exp\{-b(L-m)\}]^{-1} + c$; ED50 = m

Strain	m	b	
S288C	−0.75 (0.01)	−11.32 (0.95)	0.19 (0.02)
S288C-V291L-1	−0.84 (0.02)	−3.94 (0.29)	0.19 (0.03)
S288C-V291L-2	−0.75 (0.02)	−7.20 (0.89)	0.18 (0.04)
S288C-V291L-3	−0.63 (0.02)	−7.22 (0.85)	0.17 (0.04)
S288C-D287N	−0.82 (0.01)	−8.03 (0.61)	0.21 (0.02)
S288C-double	−0.98 (0.03)	−5.31 (0.68)	0.30 (0.05)

Ketoconazole (Supplementary Figure 5B)

Exponential model: $g = c(1-b^L)$; ED50 = $\log(0.5)/\log(b)$

Strain	ED50	b	c
S288C	−0.82 (0.12)	2.33 (0.17)	1.07 (0.03)
S288C-V291V	−0.74 (0.16)	2.56 (0.32)	1.03 (0.04)
S288C-rev	−1.01 (0.16)	1.98 (0.16)	1.07 (0.05)
S288C-V291L-1	−0.79 (0.16)	2.40 (0.29)	1.04 (0.05)
S288C-D287N	−0.95 (0.16)	2.08 (0.32)	1.02 (0.08)
S288C-double	−1.09 (0.16)	1.89 (0.22)	1.09 (0.08)

Supplementary Table 6. Growth parameters of *Saccharomyces cerevisiae* S288C and base-edited strains in the absence of an antifungal.

**Fixed effects**[1]		***F* (with *P* and denominator DF)**			
		A[2]	***B***	***R***	***M***
Residue 287		0.00 (1.0, 17)	8.00 (0.01,17)	0.42 (0.5, 19)	12.44 (0.003, 17)
Residue 291		1.63 (0.2, 17)	0.06 (0.8, 18)	3.60 (0.07, 19)	10.44 (0.005, 17)
287:291 interaction		0.07 (0.8, 17)	0.40 (0.5, 18)	0.15 (0.7, 19)	7.09 (0.02, 17)
**Mean**		0.100	1.525	0.718	8.784
**Comparison of amino-acids at critical residues**[3]					
**Amino acid**	***N***[4]	***A***	***B***	***R***	***M***
Asp287	48	0.100	1.547	0.716	8.593
Asn287	36	0.100	1.503 **	0.720	8.974
Val291	45	0.101	1.526	0.727	8.927
Leu291	39	0.099	1.524	0.709	8.640
Average SE		0.004	0.015	0.007	0.219
**Comparison of mutant strains with wild-type strain**[1]					
**Substitution**	***N***	***A***	***B***	***R***	***M***
V291V	24	0.101	1.553	0.723	8.884
D291N	21	0.101	1.499	0.731	8.971
V291L	24	0.100	1.541	0.708	8.302 *
V291L+D287N	15	0.099	1.507	0.709	8.978
Average SE		0.005	0.019	0.010	0.232
**Random effect**		**Variance component**			
Date		0.00013	0.001	0.0000	0.334
Date:Strain		0.00001	0.000	0.0003	0.066
Date:Strain:Rep		0.00129	0.287	0.0387	0.670

[1] Denominator degrees of freedom (DF): 1 in each case.

[2] Fungal growth model: OD600 = $A + B.\{1 + \exp[-R.(t-M)]\}^{-1}$; *A*: initial optical density at 600 nm (OD600 in absorbance units [AU] cm^{-1}), *B*: difference of final and initial OD600, *R*: logistic gradient of OD600 over time (AU cm^{-1} h^{-1}), *M*: time of maximum rate of growth (h). Logistic growth models were fitted to OD600 data over 20 h using the FITCURVE directive of Genstat 23rd edition.

[3] Statistical significance of comparisons of Asp with Asn at residue 287, Val with Leu at residue 291 and mutant strains with V291V dummy-edited strain: $^{+}$ 0.05 < *P* < 0.1, ** 0.01 < *P* < 0.05. Variation in growth parameters analysed by linear mixed modelling with a fixed model of Residue287 * Residue291 and a random model of Date / Strain / Replicate, where *, / and : are the crossing, nesting and interaction operators respectively. Denominator DF rounded to nearest integer. Analysis was done with Genstat 23rd edition.

[4] Number of tests.

Supplementary Table 7. Sequencing of *Erg24* gene in *Blumeria graminis* f.sp. tritici (*Bgt*) and f.sp. *hordei* (*Bgh*) and *Erg2* in *Bgt*.

a. Sequences of primers designed to amplify and sequence *Erg24* and *Erg2*. F indicates a forward primer, R a reverse primer.

Primer	Primer sequence	Target organism and gene
ERG24-F1	TGCAGCTCAGAGTGAAGTGA	*Bgt Erg24*
ERG24-F2	GCCCAAGCCATAATCCAATCT	
ERG24-R3	TGTTGTGGCCACCGTACTAT	
ERG24-F4	GGCCACCGTACTACCAAATG	*Bgh Erg24*
ERG24-R4	ACGAAAGCTCTGCGACTCTG	
ERG24-F5	CGAACTTAACCCACGTGCTC	
ERG2-F1	CCTTAGCGTCCAGGAGTCTT	*Bgt Erg2*
ERG2-R1	GCCCAGATGATGTTTGCTGG	

b. GenBank accession numbers of gene and inferred protein sequences. Mutations are relative to isolate JIW11 in *Bgt* and DH14 in *Bgh*.

Gene	Special form	Isolate	DNA sequence	Protein sequence	Amino-acid mutations
Erg24	*Bgt*	JIW11	PQ848564	TBC	–
Erg24	*Bgt*	CAW14S6303	PQ848566	TBC	V295L
Erg24	*Bgt*	96224	PQ848565	TBC	Y165F, V295L
Erg24	*Bgh*	DH14	PQ848567	TBC	–
Erg24	*Bgh*	W4	PQ848568	TBC	L77F, V295L
Erg24	*Bgh*	CC148	PQ848569	TBC	D291N
Erg2	*Bgt*	JIW11	PQ848570	TBC	–
Erg2	*Bgt*	GAP.B.2.2	PQ848571	TBC	–

Note: *Erg2* sequence for *Bgt* isolate 96224 retrieved from NCBI via accession EPQ63078.1.

Supplementary Table 8. Primers used in *Saccharomyces cerevisiae* strain S288C to test the effect of mutations detected in *Blumeria graminis* ERG24 on responses to cyclic amine antifungals.

A. Primers used in base editing to introduce single-nucleotide substitutions.

Primer name	Sequence	Function
sgRNA-guide1F	5'-CGGGTGGCGA ATGGGACTTT **TTACTTGAGT GTTTCCCCTG** GTTTTAGAGC TAGAAATAGC-3'	F primer to copy 60 mer with guide sequence in bold
sgRNA-guide1R	5'-GCTATTTCTA GCTCTAAAAC **CAGGGGAAAC ACTCAAGTAA** AAAGTCCCAT TCGCCACCCG-3'	R primer to copy 60 mer
60 mer for point mutant 1	*5'-ACTTAAGTTT ACTTCCATTC ACCTACTCAT TACAAGCGCG* ***TTACTTGAGT GTTTCACCTG***-3'	Guide 1 in bold including silent mutation in blue; mutated codon in red with target base in large type
Mutant template F1	5'-CATGATGGAT ATCACTACAG ATGGGTTTGG TTTCATGCTA GCGTTTGGTG *ACTTAAGTTT ACTTCCATTC-3*	F primer to copy 60 mer
Mutant template R1	5'-ACCCAAAAAC ATTATGGCTA ATATACCGAC AACTTTCACC CATCCCAATT CCACAGGTGA AACACTCAAG-3'	R primer to copy 60 mer
161bp repair template: mutant 1	5'-CATGATGGAT ATCACTACAG ATGGGTTTGG TTTCATGCTA GCGTTTGGTG *ACTTAAGTTT ACTTCCATTC CTACTCATTA CAAGCGCGTT* ACTTGAGTGT TTCACCTGTG GAATTGGGAT GGGTGAAAGT TGTCGGTATA TTAGCCATAA TGTTTTTGGG T-3'	
sgRNA-guide2F	5'-CGGGTGGCGA ATGGGACTTT **GAGTGTTTCC CCTGTGGAAT** GTTTTAGAGC TAGAAATAGC-3'	F primer to copy 60 mer with guide sequence in bold
sgRNA-guide2R	5'-GCTATTTCTA GCTCTAAAAC **ATTCCACAGG GGAAACACTC** AAAGTCCCAT TCGCCACCCG-3'	R primer to copy 60 mer
60 mer for point mutant 2	*5'-GTTTACTTCC ATTCACCTAC TCATTACAAG CGCGTTACTT* ***GAGTGTTTCA CCTGTGGAAT***-3'	Guide 2 in bold including silent mutation in blue; mutated codon in red with target base in large type
Mutant template F2	5'-GGATATCACT ACAGATGGGT TTGGTTTCAT GCTAGCGTTT GGTG*ACTTAA GTTTACTTCC ATTCACCTAC-3'*	F primer to copy 60 mer
Mutant template R2	5'-GAAACCCAAA AACATTATGG CTAATATACC GACAACTTTC ACCCATCCCA ATTCCACAGG TGAAACACTC-3'	R primer to copy 60 mer

160bp repair template: mutant 2	5'-GGATATCACT ACAGATGGGT TTGGTTTCAT GCTAGCGTTT GGTG*ACTTAA GTTTA***C***TTCC ATTCACCTAC TCATTACAAG CGCG*TTACTT *GAGTGTTTC***A** CCTGTGGAAT* **TGG**GATGGGT GAAAGTTGTC GGTATATTAG CCATAATGTT TTTGGGTTTC-3'	
sgRNA-guide3F	5'-CGGGTGGCGA ATGGGACTTT **ATCTGTAGTG ATATCCATCA** GTTTTAGAGC TAGAAATAGC-3'	F primer to copy 60 mer with guide sequence in bold
sg-RNA-guide3R	5'-GCTATTTCTA GCTCTAAAA**C TGATGGATAT CACTACAGAT** AAAGTCCCAT TCGCCACCCG-3'	R primer to copy 60 mer
60 mer for point mutant 3	*5'-TGG**AAG**TAAA CTTAAGT*CACCAAACGCTAG CATGAAACCA AACCC***ATCTG TAGTGATGTCCATCA*** -3'	Guide 3 in bold including silent mutation in blue; mutated codon in red with target base in large type
Mutant template F2	5'-AATTCCACAG GGGAAACACT CAAGTAA*CGC GCTTGTAATG AGTAGGTGAA TGG**AAG**TAAA CTTAAGTCAC-3'*	F primer to copy 60 mer
Mutant template R2	5'-GGATTTTACA TTTTCGATGG AGTACTAAAC GAGGAAGGTG TATTAACCAT GATGGACATC ACTACAGAT-3'	R primer to copy 60 mer
164bp repair template: mutant 3	5'-AATTCCACAG GGGAAACACT CAAGTAA*CGC GCTTGTAATG AGTAGGTGAA TGG**AAG**TAAA CTTAAGT*CAC CAAACGCTAG CATGAAACCA AACCCATCTG TAGTGAT**G**TC CATCATGGTT AATACACCTT CCTCGTTTAG TACTCCATCG AAAATGTAAA ATCC-3'	

B. Primers used to check that mutations had been introduced correctly.

Primer	Sequence 5' to 3'	Function
YscreenF1	CTTTCCTGTCTGCATCACCA	Amplify mutated region
YscreenR1	GCACTTGTGCTCATCACGTT	Amplify mutated region
YscreenR2	TGTCAAGGGAGTTTGGAACC	Amplify mutated region
YseqF	TCAACGATGCATTGGTCTTG	Sequence mutated region
YseqR	CAGCCAATCGCCAAAGTAAT	Sequence mutated region

Supplementary Table 9. Identification of residues, highlighted in red, which consistently differ between host and fungal pathogen taxa in charge, polarity or size, illustrated by the linker between transmembrane domains 6 and 7 of ERG24. Note that *Bgt*Asp277 is conserved throughout the tree of life. Other such contrasts are present in ERG24 at the active site, the binding site of NADPH and near *Bgt*Asp277.

A. Major taxa of crop plants and major taxa of fungi pathogens of crops

Outline classification						Species	Common name	Sequence ID	Sequence of Bgt274-282
Plants	Angiosperms	Eudicots	Super-asterids	Euast-erids	Campanulids	*Helianthus annuus*	Sunflower	XP_022024440.1	STWDIIAER
					Lamiids	*Capsicum annuum*	Chilli pepper	XP_016542888.2	STWDIIAER
					Ericales	*Camellia sinensis*	Tea	XP_028099757.1	STWDIIAEK
			Super-rosids	Euro-sids	Malvids	*Mangifera indica*	Mango	XP_044483840.1	STWDIIAER
					Fabids	*Cucumis melo*	Melon	KAA0043924.1	STWDIIAER
					Vitales	*Vitis vinifera*	Grapevine	XP_002283576.1	STWDIIAER
					Proteales	*Nelumbo nucifera*	Sacred lotus	XP_010278239.1	STWDIIAER
					Ranunculales	*Papaver somniferum*	Opium poppy	XP_026411861.1	STWDIIAER
		Mono-cots			Commelinids	*Triticum aestivum*	Bread wheat	XP_044423045.1	STWDIIAER
					Asparagales	*Asparagus officinalis*	Asparagus	XP_020250392.1	STWDIIAER
					Magnoliids	*Persea americana*	Avocado	KAJ8639513.1	STWDIIAER
	Gymno-sperms				Cupressaceae	*Cryptomeria japonica*	Japanese cedar (sugi)	XP_057822875.1	STWDIIAEN
					Taxaceae	*Taxus chinensis*	Chinese yew	KAH9310976.1	STWDIIAEN
Fungi	Ascomycota				Dothidiomycetes	*Zymoseptoria tritici*	Wheat Septoria blotch	XP_003853302.1	TTMDITTDG
					Eurotiomycetes	*Penicillium italicum*	Citrus green mould	KGO71811.1	TTMDVCMDG
					Leotiomycetes	*Blumeria graminis*	Wheat powdery mildew	PQ848564	TTMDITSDG
					Sordariomycetes	*Gaeumannnomyces tritici*	Take-all of wheat	XP_009229331.1	TMMDITTDG
	Basidio-mycota				Agaricomycotina	*Serpula lacrymans*	Dry rot	XP_007322902.1	TTMDITTDG
					Pucciniomycotina	*Puccinia graminis*	Wheat stem rust	KAA1101681.1	TQMDITTDG
					Ustilaginomycotina	*Ustilago tritici*	Loose smut of wheat	KAJ1022850.1	STMDITTDG

B. Humans, domesticated animals and important pathogenic fungi

Outline classification						Species	Common name	Sequence ID	Sequence of Bg274-282
Animalia					Mammalia	*Bos taurus*	Cow	NP_777047.2	TTMDITHDG
Animalia					Mammalia	*Canis lupus*	Dog	XP_038280980.1	TTMDITHDG
Animalia					Mammalia	*Homo sapiens*	Human	NP_003264.2	TTMDITHDG
Animalia					Aves	*Gallus gallus*	Chicken	NP_001383068.1	TTMDITHDG
Fungi	Dikarya	Ascomycota	Saccharomycotina			*Candida albicans*		RLP63583.1	TMIDITTDG
Fungi	Dikarya	Ascomycota	Saccharomycotina			*Candida auris*		XP_028890345.2	TMMDIATDG
Fungi	Dikarya	Ascomycota	Saccharomycotina			*Nakaseomyces glabratus*		KAJ9570472.1	TMMDITTDG
Fungi	Dikarya	Ascomycota	Pezizomycotina	Eurotiomycetes	Eurotiales	*Aspergillus fumigatus*		XP_750065.1	TTMDVIMDG
Fungi	Dikarya	Ascomycota	Pezizomycotina	Eurotiomycetes	Onygenales	*Coccidioides immitis*		KMP10110.1	GMMDITTDG
Fungi	Dikarya	Ascomycota	Pezizomycotina	Eurotiomycetes	Onygenales	*Histoplasma capsulatum*		XP_045285478.1	TTMDVIMDG
Fungi	Dikarya	Ascomycota	Pezizomycotina	Eurotiomycetes	Onygenales	*Paracoccidioides lutzii*		XP_002795907.1	TTMDMITDG
Fungi	Dikarya	Ascomycota	Pezizomycotina	Eurotiomycetes	Onygenales	*Trichophyton interdigitale*		KAF3897366.1	TMMDTTTDG
Fungi	Dikarya	Ascomycota	Pezizomycotina	Sordario-mycetes		*Fusarium falciforme*		KAJ4198337.1	TTMDITTDG
Fungi	Dikarya	Ascomycota	Pezizomycotina	Sordario-mycetes		*Sporothrix schenckii*		XP_016584084.1	TTIDITTDG
Fungi	Dikarya	Basidiomycota				*Cryptococcus neoformans*		OWT41757.1	NQMDITTDG
Fungi	Mucoromycota					*Lichtheimia corymbifera*		CDH59242.1	TTMDITTDG
Fungi	Mucoromycota					*Mucor lusitanicus*		OAD00002.1	TTMDITTDG
Fungi	Mucoromycota					*Rhizopus microsporus*		XP_023463263.1	TTMDITTDG
